# Harnessing the Power of CAR-NK Cells for Solid Tumors: Challenges, Innovations, and Future Frontiers in Immunotherapy

**DOI:** 10.34133/cancomm.0023

**Published:** 2026-04-10

**Authors:** Mengchao An, Jiayao Yan, Baorui Liu, Qin Liu

**Affiliations:** The Comprehensive Cancer Center, Nanjing Drum Tower Hospital & Group’s Suqian Hospital, Affiliated Hospital of Medical School, Nanjing University, Nanjing, China.

## Abstract

Solid tumors remain a formidable challenge in cancer therapy, often evading even the most advanced immunotherapies. Natural killer (NK) cells, cytotoxic innate lymphocytes capable of recognizing and eliminating tumor cells without prior antigen sensitization, have emerged as a compelling alternative to T cells in adoptive cell therapy. Compared to chimeric antigen receptor (CAR)-T cells, CAR-engineered NK cells offer distinct advantages, including a substantially reduced risk of graft-versus-host disease (GvHD) and cytokine release syndrome (CRS). These features enable the development of “off-the-shelf” allogeneic cell products with improved safety and accessibility. Early clinical studies of CAR-NK cells have demonstrated encouraging efficacy in hematological malignancies alongside an excellent safety profile, fueling enthusiasm to extend this approach to solid tumors. However, the efficacy of CAR-NK cell therapy against solid tumors is limited by multiple barriers, including the immunosuppressive tumor microenvironment, poor infiltration, and persistence of NK cells in tumor tissues, heterogeneity of tumor antigen expression leading to immune escape, and the potential for NK cell dysfunction or exhaustion in chronic tumor settings. To overcome these obstacles, innovative engineering strategies are being developed. Approaches include armoring CAR-NK cells to resist tumor-induced immunosuppression, enhancing their trafficking and persistence, designing multi-antigen-targeted receptors, and incorporating built-in safety switches. This review highlights CAR-NK antitumor mechanisms, examines key challenges in solid tumor applications, and discusses cutting-edge advances and combination strategies aimed at unlocking the full therapeutic potential of CAR-NK cells. By addressing these challenges, CAR-NK cell therapy could open a new frontier in solid tumor immunotherapy.

## Introduction

Solid tumors account for the majority of cancer-related deaths, presenting unique and severe challenges to immunotherapy. By contrast, chimeric antigen receptor (CAR)-T therapies targeting CD19 or BCMA have delivered transformative and durable responses in relapsed or refractory B cell malignancies [1–3]. Importantly, current data suggest that long-term CAR-T persistence is closely linked to sustained disease control in B cell leukemias, whereas in aggressive B cell lymphomas durable remissions can be achieved even when CAR-T cells become undetectable over time [2,4], suggesting that CAR-T persistence may not be uniformly required for durable clinical benefit across disease contexts. Despite this breakthrough in certain hematological malignancies, no CAR-T therapy has yet been approved for solid tumors, highlighting the many limitations imposed by the tumor microenvironment (TME) on the efficacy of immune effector cells.

Natural killer (NK) cells, a crucial component of innate immunity, can rapidly recognize and eliminate abnormal cells, including virus-infected cells and transformed tumor cells [5]. Unlike αβ T cells, in which each clone recognizes a specific peptide antigen presented by a major histocompatibility complex (MHC) molecule via its clonally rearranged T cell receptor (TCR), NK cells integrate signals from germline-encoded activating and inhibitory receptors and can target tumor cells without prior antigen sensitization [6]. In addition, NK cells lack a conventional surface TCR and have a unique cytokine secretion profile, features that are associated with a much lower incidence of severe cytokine release syndrome (CRS) and negligible risk of graft-versus-host disease (GvHD) [5–7]. These safety advantages provide a feasible pathway for developing allogeneic or cell line-derived “off-the-shelf” CAR-NK cell products [8]. As a result, NK cells are emerging as a highly attractive platform for tumor immunotherapy.

Although CAR-NK therapy has shown promising prospects in hematological malignancies, its application in solid tumors faces greater challenges [7,9]. Beyond their more complex physical and immune barriers, solid tumors encompass biologically distinct entities—such as prostate cancer, melanoma, and colorectal carcinoma—with divergent mutational landscapes and TMEs that cannot be regarded as equivalent [10,11]. Nonetheless, many solid tumors share convergent barriers to cellular immunotherapy, including immunosuppressive TMEs, antigen heterogeneity, dense extracellular matrix (ECM), and cancer-associated fibroblasts (CAFs), which collectively limit the infiltration, persistence, and tumor cell clearance capabilities of NK cells [9,12]. In recent years, innovative strategies have been developed to enhance the efficacy of NK cells, including genetic engineering with CARs and cytokine cassettes, combination with immune checkpoint inhibitors (ICIs), and infusion of NK cells activated and expanded by cytokines such as interleukin-15 (IL-15) [13,14]. These approaches aim to overcome barriers such as poor infiltration, functional exhaustion, and immune suppression in solid tumors.

This review systematically outlines the status of CAR-NK cell immunotherapy for solid tumors, focusing on key challenges and cutting-edge solutions being developed to overcome them. We first describe how NK cells and CAR-NK cells recognize and eliminate tumor cells. We then examine the primary obstacles limiting the efficacy of CAR-NK cells in solid tumors. Next, we highlight engineering strategies designed to enhance the functionality, persistence, homing ability, and safety of CAR-NK cells. Furthermore, we discuss the synergistic effects of CAR-NK cells with other therapeutic modalities. Finally, we look ahead to emerging technologies and future directions, with the goal of providing actionable design principles and translational pathways for next-generation CAR-NK cell therapies. Our aim is to facilitate more durable and broad clinical benefits from CAR-NK cells while ensuring safety.

## Mechanisms of CAR-NK Antitumor Activity

NK cells, unlike αβ T lymphocytes whose clonally rearranged TCRs recognize peptide antigens presented by MHC molecules, rely on integrating signals from germline-encoded activating and inhibitory receptors [15]. Tumors evade T cell surveillance by down-regulating MHC-I (“missing-self”) while up-regulating stress-induced ligands for NK activating receptors such as NKG2D and the natural cytotoxicity receptors (e.g., NKp30 and NKp46), providing broader coverage in the face of antigen heterogeneity [9,15]. The resulting imbalance—loss of inhibitory signaling coupled with increased activation—triggers NK cell activation and target cell elimination.

Once activated, NK cells employ multiple cytotoxic mechanisms to eradicate targets. A primary mode is granule-mediated killing: NK cells release perforin, which forms pores in the target cell membrane, allowing entry of granzymes that trigger caspase-dependent apoptosis [15,16]. NK cells can also induce cell death via the “death receptor” pathway by up-regulating Fas ligand (FasL/CD95L) and tumor necrosis factor (TNF)-related apoptosis-inducing ligand (TRAIL); engagement of Fas or TRAIL receptors on the target cell activates caspase-8 and downstream apoptotic cascades [16,17]. NK cells can also mediate antibody-dependent cellular cytotoxicity (ADCC) through the FcγRIIIA (CD16A) receptor: Binding of CD16A to IgG-coated target cells triggers immunoreceptor tyrosine-based activation motif (ITAM)-dependent signals that culminate in target lysis [5,15]. In addition, NK cells secrete a variety of cytokines and chemokines, which not only directly inhibit tumor growth but also remodel the TME and recruit other immune effector cells to the antitumor response [5,15]. However, in vivo imaging in interferon-γ (IFN-γ) reporter mice has shown that intratumoral NK cells contribute relatively little IFN-γ compared with T cells [18], and a head-to-head comparison of human CAR-NK and CAR-T cells demonstrated that CAR-NK cells secrete markedly less IFN-γ despite retaining potent cytotoxicity [19]. These findings suggest that CAR-NK therapies may rely more on direct cytotoxicity, with a comparatively smaller contribution from IFN-γ-driven remodeling of the TME. Against this backdrop of innate recognition and effector functions, CAR engineering provides a way to redirect NK activity toward defined tumor antigens.

CARs endow NK cells with targeted tumor specificity by grafting an antigen-recognition domain onto the cell surface. CARs typically consist of an extracellular tumor antigen-binding single-chain variable fragment (scFv) joined via a hinge and transmembrane region to intracellular signaling domains [20]. Early-generation CAR designs, originally developed for T cells, used the CD3ζ chain with its ITAM motifs, alone or with T cell costimulatory domains [20]. In an NK cell, engagement of a CD3ζ-based CAR recruits Syk/ZAP70 family kinases to the ITAMs, initiating signaling cascades analogous to native NK activation, including phospholipase C-γ (PLC-γ) activation, Ca^2+^ mobilization, and NFAT nuclear translocation that drives cytokine gene transcription [20–22]. These signals converge on the NK cell’s cytoskeletal machinery, leading to perforin/granzyme exocytosis and target cell killing, mirroring the physiological NK response [6].

Importantly, preclinical studies suggested that simply transplanting T cell-derived CAR architectures into NK cells may not fully harness NK-intrinsic signaling, motivating designs that incorporate NK-specific costimulatory modules [6,23,24]. NK cells natively express several adaptor proteins—CD3ζ, FcεRIγ, DAP12, and DAP10—that associate with activating receptors [6]. DAP12 contains ITAMs like CD3ζ and signals via ZAP70/Syk, whereas DAP10 carries a YxxM immunoreceptor tyrosine tail motif that activates phosphatidylinositol 3-kinase (PI3K)–Akt and Vav1 pathways independently of Syk/ZAP70 [21]. In parallel, 2B4 (CD244) has an immunoreceptor tyrosine-based switch motif and signals through the SLAM-associated protein adaptor, providing a complementary co-activation pathway [25]. Incorporating such domains into CAR constructs allows synergistic activation of NK cells. For example, an optimized CAR containing DAP10 and 2B4 signaling domains alongside CD3ζ elicited markedly stronger NK cell cytotoxicity than a conventional CAR using only T cell-derived domains [23]. In a recent study, Acharya et al. [24] systematically compared costimulatory domains in CAR-NK cells and showed that CD28 costimulation engaging the LCK–CD3ζ–ZAP70 axis enhanced proximal signaling and antitumor activity compared with conventional 4-1BB- or T cell-oriented designs. Together, these NK-tuned CAR designs couple synthetic antigen recognition to the endogenous NK effector machinery described above, thereby providing a mechanistic basis for CAR-NK antitumor activity.

## Challenges for CAR-NK Cells in Solid Tumors

**Fig. 1.**
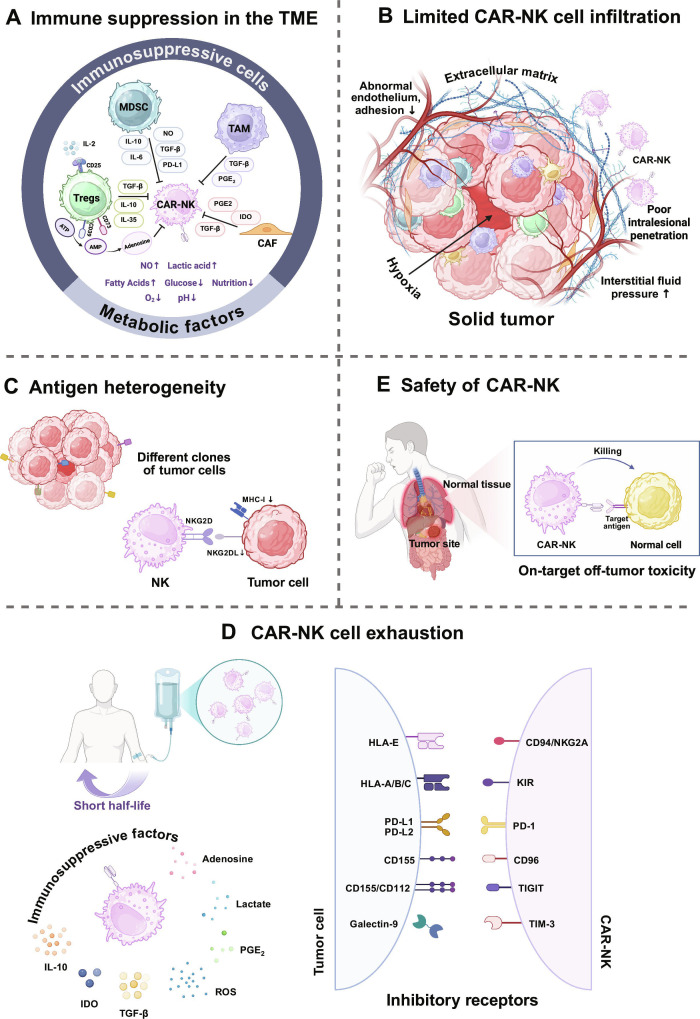
Challenges for CAR-NK cells in solid tumors. (A) Cellular and metabolic suppressors in the TME restrain CAR-NK activity. Solid tumors create an immunosuppressive microenvironment by expanding Tregs, MDSCs, TAMs, and CAFs. Hypoxia and metabolic derangements further impede CAR-NK cell effector function. (B) Multiple barriers restrict the trafficking and penetration of CAR-NK cells. CAF-driven dense stroma increases tissue stiffness and interstitial fluid pressure. Hypoxia aggravates vascular abnormalities and down-regulates endothelial adhesion molecule expression, impairing CAR-NK cell infiltration into tumors. (C) Antigen heterogeneity. Solid tumors are composed of subclones with distinct antigen profiles and variable target density, and some clones are antigen negative. NK cells can recognize missing-self through MHC-I down-regulation, yet tumors evade this pathway by down-regulating NKG2D ligands, thereby suppressing innate NK activation. (D) CAR-NK exhaustion in solid tumors (bottom panel). Persistence after infusion is often brief. Chronic antigen exposure and TME suppression induce exhaustion with inhibitory receptor up-regulation and impaired function. Metabolic and nutrient limits reinforce this program and sustain dysfunction. (E) Safety (middle right panel). When target antigens are shared with normal tissues, recognition can cause on-target off-tumor injury, with risk determined by antigen distribution and expression level. Figures were created with BioRender.com. CAF, cancer-associated fibroblast; ECM, extracellular matrix; IDO, indoleamine 2,3-dioxygenase; KIR, killer cell immunoglobulin-like receptor; MDSC, myeloid-derived suppressor cell; MHC-I, major histocompatibility complex class I; NKG2DL, NKG2D ligand; NO, nitric oxide; ROS, reactive oxygen species; TAM, tumor-associated macrophage; TGF-β, transforming growth factor-β; TME, tumor microenvironment; Tregs, regulatory T cells.

Compared with hematological malignancies, solid tumors present greater challenges for CAR-NK cell therapy. Within a complex, often immunosuppressive TME, CAR-NK cells must not only target tumor cells but also overcome various immunosuppressive factors and physical barriers (Fig. 1).

### Immune suppression in the TME

The TME is characteristically immunosuppressive and remains a principal barrier to adoptive cell therapies in solid tumors [26]. It comprises diverse immunosuppressive cells and matrix components, including myeloid-derived suppressor cells (MDSCs), regulatory T cells (Tregs), tumor-associated macrophages (TAMs), endothelial cells, and CAFs, all embedded within a dense ECM [13] (Fig. 1A).

Tregs are key regulators of immune homeostasis that prevent autoimmunity but can markedly suppress NK-mediated antitumor responses in the TME [27–29]. As early as 1999, studies have shown that depletion of CD25^+^CD4^+^ T cells unmasks potent antitumor immunity, establishing Tregs as key mediators of tumor immune evasion [30]. One primary mechanism by which Tregs suppress NK cells is through the secretion of transforming growth factor-β (TGF-β), which attenuates NK cytotoxicity by constraining mechanistic target of rapamycin(mTOR)-dependent metabolic programs [27,31]. TGF-β can also inhibit the expression of multiple activating receptors on tumor-infiltrating NK cells, including NKG2D [28,31]. In non-small cell lung cancer (NSCLC), TGF-β was identified as a primary factor suppressing NK cells [29]. Furthermore, Tregs participate in NK cell inhibition through adenosine triphosphate (ATP) metabolism. ATP enriched in the TME can establish a feedback loop with CD39 and CD73 expressed on Tregs, sustaining adenosine production and immune suppression [32–34]. Competition for IL-2, which is essential for NK cell survival and function, adds another layer of inhibition [35]. Ipilimumab, which targets the Treg-overexpressed receptor cytotoxic T lymphocyte antigen 4 (CTLA-4), can restore NK cell-mediated ADCC in head and neck cancer (HNC) [36].

MDSCs are a heterogeneous population derived from immature myeloid precursors, which expand pathologically in tumors and exert potent immunosuppression [37]. Within the TME, membrane-bound TGF-β on MDSCs down-regulated NKG2D and IFN-γ, impairing NK cell activation [38]. In hepatocellular carcinoma (HCC), MDSCs further impeded NK antitumor function through the NKp30 pathway [39]. Additionally, MDSC-derived nitric oxide (NO) disrupted Fc receptor-mediated NK cell responses [40], while arginase and indoleamine 2,3-dioxygenase (IDO) amplified suppression [41,42]. Accordingly, inhibiting MDSC proliferation, recruitment, or immunosuppressive functions may enhance the efficacy of NK cell-based therapy, including CAR-NK cells. Targeting C–X–C chemokine receptor 1 (CXCR1) and CXCR2 to inhibit MDSC aggregation has been shown to enhance NK cell antitumor activity in preclinical HNC models [43]. Vascular endothelial growth factor (VEGF) pathway inhibitors, such as bevacizumab, have been associated with reductions in circulating MDSC levels in patients with NSCLC [44]. In addition, employing specific chemotherapeutic agents such as doxorubicin and gemcitabine [45,46], blockade of MDSC-derived prostaglandin E_2_ (PGE_2_) [47], and treatment with all-trans retinoic acid have been reported to inhibit MDSCs and may thereby help relieve NK cell functional inhibition [48].

TAMs play an important role in immune evasion [49]. Several studies indicate that TAMs induced NK cell dysfunction by secreting TGF-β [50]. In HCC, CD48-expressing TAMs directly engaged the 2B4 receptor on NK cells, delivering inhibitory signals that attenuate NK cytotoxicity [51]. TAMs also secrete the immunosuppressive chemokines CCL5 and CCL22, recruiting Tregs into the TME and further suppressing the antitumor effect of NK cells [49]. To mitigate TAM-mediated immunosuppression, Eisinger et al. [52] employed antibodies targeting the TAM scavenger receptor MARCO to restore NK cell-mediated killing of human melanoma cells.

CAFs directly suppress NK cell activity, most notably via abundant secretion of TGF-β [53]. Tumor-conditioned CAFs from melanoma, colorectal cancer (CRC), or HCC secreted PGE_2_ and IDO, reprogramming NK cells toward a hyporesponsive state with downshifted activating phenotypes [54–56]. CAFs derived from endometrial carcinoma down-regulated the DNAM-1 ligand CD155 on tumor cells, further impairing NK cell activity [57]. Functionally, various strategies to target or reprogram CAFs have been demonstrated to alleviate NK dysfunction within the TME in pancreatic ductal adenocarcinoma and CRC [58,59]. In parallel, CAF-driven ECM remodeling forms a hard, dense matrix that acts as a physical barrier to NK and CAR-NK infiltration, as detailed below.

The metabolic landscape of the TME further constrains the efficacy of NK cells. The rapid tumor growth and abnormal vasculature induce hypoxia, which reprograms NK cells and impairs their effector functions [60]. mTOR-coupled stress responses that drive mitochondrial injury, down-regulation of activating receptors, and autophagic degradation of granzyme B represent core mechanisms underlying hypoxia-induced NK dysfunction [61–63]. In parallel, adenosine, the terminal product of extracellular ATP catabolism, accumulates in the TME. Upon binding to A2A adenosine receptors on NK cells, it can trigger adenosine 3′,5′-monophosphate (cAMP)-dependent inhibitory cascades, causing potent suppression of NK activation and cytotoxicity [64,65].

Overall, these cellular and metabolic suppressive programs in the TME can blunt NK effector function and are likely to similarly limit CAR-NK activity in solid tumors. This underscores the need for CAR-NK designs and combinations that actively counter dominant inhibitory cues to achieve durable antitumor efficacy.

### Limited CAR-NK cell infiltration

A central bottleneck for CAR-NK therapy in solid tumors is the inability of effector cells to efficiently enter and reside within the TME (Fig. 1B). The biochemical and biomechanical features of solid tumors together can raise the thresholds for vascular extravasation and interstitial migration, markedly diminishing NK cell delivery to tumors [66–68]. The tumor stroma comprises dense ECM, basement membranes, CAFs, and endothelial cells such that NK cells migrating to the lesions are often surrounded at the periphery by a high-stiffness matrix [67,68]. CAFs, major contributors to ECM deposition, secrete TGF-β to promote tumor growth, drive epithelial-to-mesenchymal transition, and suppress immune infiltration [67,69]. Moreover, extensive fibrillar collagen deposition and up-regulation of collagen-processing enzymes result in a highly stiff tumor matrix. This is accompanied by hypoxia and elevated interstitial fluid pressure, which further impede immune cell infiltration [67,70–72].

Hypoxia aggravated vascular abnormalities and down-regulated endothelial adhesion molecules, creating physical barriers to leukocyte extravasation and directly limiting the efficacy of CAR-NK and other immunotherapies [73]. At the molecular level, hypoxia-inducible factor (HIF) signaling induced by hypoxia up-regulates pro-angiogenic factors and weakens the interaction between immune cells and the vascular wall, thereby diminishing immune infiltration [74,75]. Chemokine axes are equally pivotal, as NK recruitment to solid tumors depends on receptor-ligand alignment [76], with CXCR3 playing a particularly important role [77]. In many TMEs, however, NK cells exhibited insufficient chemokine receptor expression or encountered paucity of the corresponding ligands, resulting in mistargeting and poor tissue perfusion [78].

In summary, these physical, vascular, and chemokine-related abnormalities converge to disrupt NK cell trafficking and distribution in solid tumors. Because CAR-NK cells depend on similar homing and migration pathways, such barriers are anticipated to limit their accumulation at tumor sites and thereby curb therapeutic efficacy.

### Antigen heterogeneity

Antigen heterogeneity and antigen loss are major barriers to CAR-NK efficacy in solid tumors (Fig. 1C). Solid tumors are mosaics of subclones that express distinct antigens or, in some cases, completely lack target expression [79]. Many tumors further evade conventional T cell surveillance by down-regulating or losing MHC-I, creating niches for antigen- or MHC-low variants; NK cells can target such “missing-self” clones, but tumors can further down-regulate NKG2D ligands to counteract this pathway, thereby evading NK cell-mediated lysis [80,81]. Moreover, intratumor diversity results in nonoverlapping antigen expression profiles, making single-antigen CAR-NK products unlikely to cover the full malignant repertoire [82]. Antigen escape mechanisms further exacerbate this situation, with tumor cells undergoing epigenetic reprogramming or up-regulating compensatory survival pathways to evade immune attack [83]. Consequently, targeting a single tumor-associated antigen (TAA) often eliminates only a fraction of disease, while antigen-negative or low-antigen clones persist and drive relapse [84].

### NK cell exhaustion

A major clinical hurdle for NK cell immunotherapy is their brief in vivo persistence after infusion, typically detectable for only a few weeks [85,86]. Under the combined pressure of chronic antigen exposure and TME-derived suppression, NK cells progressively acquire an exhaustion phenotype characterized by increased expression of inhibitory receptors such as NKG2A, PD-1, TIGIT, and TIM-3 in response to tumor ligands, together with sustained exposure to suppressive factors (e.g., TGF-β, IL-10, and PGE₂), which collectively drive functional fatigue (Fig. 1D) [87–90]. This state is characterized by diminished cytokine secretion, impaired degranulation, and reduced proliferative capacity, ultimately limiting persistence and antitumor efficacy [88–90]. In addition, metabolic and nutritional limitations together with soluble suppressive mediators further exacerbate exhaustion, forming a niche that sustains NK dysfunction [91]. Overall, exhaustion is a dominant barrier to NK-based therapies in solid tumors, and similar exhaustion programs are expected to constrain the persistence and efficacy of CAR-NK cells, making prevention or reversal of this dysfunctional state a central focus of current research.

### Safety

Safety remains the central and durable concern for CAR-NK and other immunotherapies. The efficacy of CAR-NK cell therapies depends largely on antigen selection (Fig. 1E). The ideal target is highly and stably expressed on tumor cells, minimally on essential normal tissues, and broadly expressed across lesions, minimizing the risk of on-target off-tumor toxicity [92,93]. Notably, HER2 and mesothelin (MSLN) are attractive in multiple solid tumors, yet display low-level expression in certain normal tissues, underscoring the need for precise targeting [94,95]. Compared with CAR-T, CAR-NK cells have generally shown shorter in vivo persistence, which may reduce the likelihood of sustained on-target off-tumor damage to normal organs [96]. Although GvHD and CRS are generally infrequent with CAR-NK, transient and reversible hematological toxicity may still occur [7,97]. Therefore, rigorous target validation and safety switches are essential to confine potential toxicities while preserving antitumor potency.

## Engineering Strategies to Improve CAR-NK Cell Therapy for Solid Tumors

### Overcoming immunosuppression in TME

**Fig. 2.**
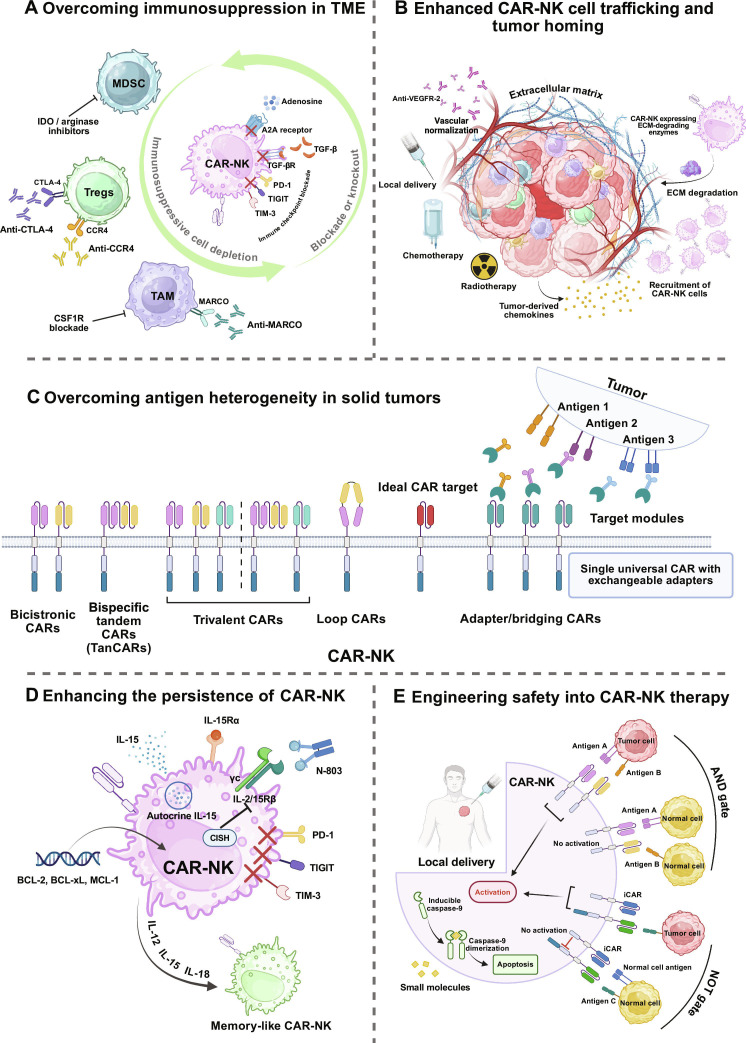
Engineering strategies to improve CAR-NK cell therapy for solid tumors. (A) Overcoming immunosuppression in the tumor microenvironment. Eliminating or reprogramming suppressive compartments and armoring CAR-NK cells against dominant checkpoints and metabolic stress can further enhance efficacy in solid tumors. (B) Multimodal strategies enhance CAR-NK trafficking and intratumoral penetration. Vascular normalization with anti-VEGF/VEGFR2 creates a transient window for entry and relieves hypoxia. Chemotherapy and radiotherapy remodel the niche, increase chemokine cues and adhesion, and can up-regulate stress ligands. Locoregional delivery enriches CAR-NK cells at disease sites while limiting systemic exposure. Tumor-derived chemokines recruit receptor-matched CAR-NK cells to the lesion. CAR-NK engineered with ECM-degrading enzymes dismantle desmoplastic matrix to expand intratumoral penetration. (C) Overcoming antigen heterogeneity with multi-target CAR designs. The above strategies broaden tumor coverage across non-overlapping antigen profiles and heterogeneous antigen density while preserving flexible control of specificity. (D) Intrinsic engineering and cytokine support enhance CAR-NK fitness. CAR-NK cells are equipped with IL-15 support through autocrine or membrane-bound IL-15 and through the IL-15 superagonist N-803. Blocking or knocking out inhibitory receptors including PD-1, TIGIT, and TIM-3 restores activation in suppressive niches. Up-regulation of prosurvival molecules BCL-2, BCL-xL, and MCL-1 further extends persistence. Priming with IL-12, IL-15, and IL-18 generates cytokine-induced memory-like CAR-NK cells with heightened durability and effector function. (E) Logic-gated and safety-controlled CAR-NK. Locoregional delivery further restricts CAR-NK activity to the tumor bed and reduces systemic exposure. An AND gate requires cis co-engagement of antigens A and B on the same target cell to trigger activation. A NOT gate uses an iCAR that recognizes a normal tissue antigen and delivers inhibitory signaling, preventing killing when the off-tumor antigen is present. An inducible suicide switch iCasp9 is activated by a small-molecule to dimerize caspase-9 and rapidly ablate CAR-NK cells. Figures were created with BioRender.com. CAR, chimeric antigen receptor; CCR4, CC chemokine receptor 4; CSF1R, colony-stimulating factor 1 receptor; ECM, extracellular matrix; iCasp9, inducible caspase-9; IDO, indoleamine 2,3-dioxygenase; MDSC, myeloid-derived suppressor cell; N-803, IL-15/IL-15Rα superagonist N-803; TAM, tumor-associated macrophage; TME, tumor microenvironment; Tregs, regulatory T cells; VEGF, vascular endothelial growth factor; VEGFR2, vascular endothelial growth factor receptor 2.

Enhancing CAR-NK efficacy in solid tumors hinges on alleviating or reversing the immunosuppressive milieu of the TME (Fig. 2A). The primary principle is to deplete or reprogram suppressive compartments, including TAMs, MDSCs, Tregs, and CAFs, thereby creating a conducive microenvironment for CAR-NK infiltration, survival, and killing. For MDSCs, preclinical studies showed that either depletion or blockade of their dominant suppressive programs can restore NK cell activity and improve outcomes [98]. For instance, blocking reactive oxygen species (ROS) production or arginase activity has been reported to relieve MDSC-mediated NK dysfunction and has been proposed as a rational combinatorial strategy alongside CAR-NK therapy [98,99]. In the macrophage compartment, colony-stimulating factor 1 receptor (CSF1R) inhibition depleted TAMs and reduced protumor myeloid subsets, which can indirectly support antitumor immune responses [100]. CAF-targeting strategies have shown promise in preclinical CAR-T models and are increasingly discussed as templates for CAF-directed CAR-NK platforms, although such applications remain largely conceptual at present [101,102]. Collectively, these interventions may help remodel the TME from a suppressive to a permissive niche, with the potential to enhance CAR-NK trafficking, persistence, and cytotoxic potency.

TGF-β impinges on virtually every aspect of NK antitumor function, including cytokine production, degranulation, cellular metabolism, and mTOR signaling, thereby enforcing dysfunction in solid tumors [91,103–105]. For CAR-NK therapy, a first strategy is to hard-wire resistance to this axis. Incorporating a dominant-negative TGF-βR2, or CRISPR-mediated *TGFBR2* knockout, in CAR-NK platforms may preserve effector function under high TGF-β and enhance antitumor activity in TGF-β-rich TMEs [106]. An ongoing phase I trial of TGF-βR2/*NR3C1*-double-deficient NK cells in relapsed/refractory glioblastoma (NCT04991870) further illustrates the clinical feasibility of dual resistance modules and provides a conceptual blueprint for analogous CAR-NK designs. Beyond receptor editing, paracrine antagonism of TGF-β within the TME is emerging as a complementary tactic. CAR-NK cells engineered to secrete a TGF-β receptor-inhibitory peptide (UP01a) locally neutralized TGF-β signaling, reducing downstream immunosuppressive mediators, increasing intratumoral NK infiltration, and improving antitumor efficacy [107]. Shin et al. [108] devised a self-activating CAR-NK that, upon tumor engagement, releases the designer peptide P6 to block TGF-β1 signaling; disruption of the SMAD2/3 cascade in this setting restores CAR-NK metabolic programs and cytotoxicity.

To counter lactate, adenosine, ROS, and hypoxia in the TME, constructing environment-sensing, suppression-resistant CAR-NK cells is an emerging direction to strengthen efficacy in solid tumors. On one front, overexpression of antioxidant enzymes such as PRDX-1 improved CAR-NK tolerance and cytotoxic performance under oxidative stress in preclinical models [109]. In parallel, dampening hypoxia-responsive HIF-1α signaling in NK cells has been reported to augment NK-mediated antitumor activity [110]. Juillerat et al. [111] fused the oxygen-dependent degradation domain of HIF-1α to the CAR, yielding an oxygen-sensitive, self-decision-making receptor (HIF-CAR). Under normoxia, HIF-CAR is rapidly ubiquitinated and degraded, whereas in hypoxia it is stabilized and remains fully signaling, thereby restricting cytotoxic activation to low-oxygen niches within the TME. This modular control logic could in principle be ported to CAR-NK platforms to restrict receptor signaling to hypoxic niches, with the aim of enhancing intratumoral activity and persistence. On a complementary front, targeting adenosinergic suppression offers another lever. In NK cells, A2AR signaling restrained maturation and effector function, and its disruption improved NK-mediated tumor control in preclinical models [112]. Engineering CAR-NK cells to resist adenosine is therefore an appealing strategy for next-generation CAR-NK. Conceptual work suggests that up-regulating amino acid transporters to improve nutrient uptake could buffer CAR-NK cells against TME nutrient deprivation and preserve effector function [113].

### Enhanced CAR-NK cell trafficking and tumor homing

To enhance intratumoral delivery of CAR-NK cells, chemokine-axis engineering can be used to align receptor expression with the tumor chemokine milieu and thereby improve trafficking and local accumulation. In HCC models, CXCR2-armed CAR-NK cells showed enhanced migration toward tumor-derived chemokines and improved antitumor activity [114]. In preclinical models, EGFRvIII-CAR-NK cells overexpressing CXCR4 showed improved homing to glioblastoma, whereas CXCR1-expressing NKG2D-CAR-NK cells infiltrated ovarian cancer nodules more efficiently [115,116]. Given intertumoral heterogeneity in chemokine repertoires and kinetics, exogenous intratumoral chemokine augmentation has been explored for adoptively transferred NK cells [6]. CCR5-engineered NK cells preferentially homed to tumors infected with CCL5-modified oncolytic vaccinia, enhancing trafficking and efficacy [117]. Likewise, an NK-recruiting antibody fusion protein (NRP-body) that deposits CXCL16 within tumors established a local gradient and markedly augmented NK homing and infiltration [118]. Together, these NK-based preclinical studies support a modular approach in which chemokine receptor engineering is paired with controlled chemokine delivery, a principle that could be applied to CAR-NK products to improve homing in clinically relevant solid tumor models.

The dense ECM of solid tumors constitutes a major barrier to NK cell infiltration. One strategy is to equip CAR-NK cells with ECM-degrading enzymes to locally dismantle matrix components and improve intratumoral penetration. In CAR-T models, co-expression of heparinase (HPSE) enhanced degradation of fibrotic stroma, improved infiltration of anti-GD2 CAR-T cells, and augmented antitumor activity [119]. Consistent with this, NK cells bearing membrane-tethered, catalytically active HPSE showed superior intratumoral accumulation and cytotoxicity compared to unmodified NK counterparts [120]. Together, these CAR-T and NK data provide a mechanistic blueprint for ECM-remodeling CAR-NK products that co-express HPSE or hyaluronidase to achieve controlled local degradation of stromal barriers and deeper parenchymal access. However, ECM degradation also carries potential risks of off-tumor tissue injury and tumor dissemination. These risks may be mitigated by restricting enzyme activity to the tumor, using controllable expression systems, and confining expression to defined time windows. With careful spatiotemporal control, local matrix remodeling could directly breach the physical barriers of the TME and complement chemokine-axis engineering.

Abnormal tumor vasculature also limits the extravasation of effector cells. Low-dose anti-angiogenic therapy has been shown in preclinical models to transiently normalize vessels and improve perfusion, thereby supporting more effective trafficking of immune cells to tumors [121,122]. Agents such as VEGF inhibitors or VEGFR/VEGFR2 tyrosine kinase inhibitors (TKIs) can remodel vascular architecture and relieve hypoxia, which indirectly promotes lymphocyte infiltration [123]. In glioblastoma models, anti-VEGF antibodies increased CAR-T penetration and distribution while delaying tumor growth [124]. Although shown with CAR-T cells, the same vascular-normalization principles could be exploited to improve CAR-NK trafficking. Dose and timing require careful optimization, as excessive blockade of angiogenesis can over-prune the vascular bed, whereas moderate normalization creates a temporal window that could, in principle, enhance entry of CAR-NK cells [122]. Conceptually, vascular targeting may act synergistically with chemokine- or integrin-based homing strategies since improved vascular function enables engineered CAR-NK cells to follow guidance cues into the tumor.

Beyond systemic administration, locoregional delivery is being explored as a promising strategy to enrich CAR-NK cells within tumor sites. By bypassing intravascular attrition and peripheral clearance, this approach increases on-target accumulation while reducing off-tumor exposure. In glioblastoma xenografts, local injection of CAR-NK cells markedly delayed tumor growth and prolonged survival [125]. The first clinical study of CAR-NK in solid tumors treated 3 patients with metastatic CRC using locoregionally delivered NKG2D CAR-NK cells; intraperitoneal and percutaneous infusions were feasible and well tolerated, with evidence of reduced malignant ascites and metabolic responses in some lesions [126]. In addition, intra-arterial hepatic delivery of NK cells combined with cetuximab for gastrointestinal liver metastases demonstrated a favorable safety profile and signs of activity in a phase I study, supporting the feasibility of locoregional NK cell infusion strategies that could be adapted for future CAR-NK products [127].

Radiotherapy (RT) and chemotherapy can serve as infiltration enablers. In preclinical models, RT has been shown to enhance CAR-NK trafficking and antitumor activity by remodeling the TME and inducing NKG2D ligands on tumor cells [114,128]. Low-dose or metronomic chemotherapy can transiently deplete Tregs and modulate MDSCs, thereby relieving immunosuppression and potentially creating a more permissive window for CAR-NK homing and function [129,130].

Adjunct strategies can further improve infiltration. Decorating NK cells with tumor-penetrating peptides such as iRGD leverages binding to vascular integrins and neuropilin-1, thereby enhancing parenchymal entry [131]. Experimentally, iRGD-modified NK cells penetrated more deeply into multicellular tumor spheroids and achieved superior tumor control in mouse models [132,133]. Although these studies were performed with NK cells, they provide a modular blueprint for incorporating tumor-penetrating peptides into CAR-NK products to further enhance intratumoral delivery in solid tumors.

Together, these approaches are designed to improve directional homing, transendothelial trafficking, and intratumoral retention of CAR-NK cells, with the aim of sustaining antitumor activity (Fig. 2B). Collectively, such multidimensional optimization may help to alleviate key physical and molecular barriers imposed by the TME and could bring CAR-NK therapies closer to realizing their therapeutic potential in solid tumors.

### Overcoming antigen heterogeneity in solid tumors

Antigen heterogeneity and antigen loss are major barriers to CAR-NK efficacy in solid tumors. Multi-target design improves the completeness of tumor clearance, and the same principle applies to CAR-NK platforms [134–136]. Engineering options include tandem CARs (TanCARs) that fuse 2 or more antigen-binding domains into a single receptor and co-expression of multiple independent CARs within the same cell (Fig. 2C). These designs implement OR logic, in which recognition of any target antigen triggers activation, and AND logic, which requires cooperative recognition, thereby broadening antigen coverage, lowering escape, and reducing off-tumor risk while maintaining specificity [134–136]. Proof-of-concept studies support this strategy. The bispecific CAR-NK92 targeting CD19 and BCMA efficiently lysed single-antigen and dual-antigen targets as well as primary tumor cells in preclinical models [137]. In lung cancer models, CAR-NK cells dually targeting PD-L1 and MICA/B markedly suppressed tumor growth [138]. Anti-CD19/CD22 CAR-NK has entered clinical evaluation for relapsed and refractory B cell lymphoma (NCT03824964), illustrating the feasibility and translational promise of multi-specific CAR-NK products. Geometric optimization can further tighten control of antigen escape. Loop CARs arrange 2 antigen-binding domains in a looped spatial configuration through linker engineering, improving epitope pairing, receptor clustering, and signal transduction. In preclinical studies of acute myeloid leukemia (AML), CD33-MSLN Loop CAR-iNK showed stronger antigen-specific cytotoxicity, enhanced cytokine release and degranulation, and markedly prolonged survival in xenograft models [139]. These data indicate that coordinated optimization of receptor spatial geometry and signaling can narrow the antigen escape window while preserving safety.

Adaptor and bridging molecule CARs have been developed to achieve multi-antigen targeting. In these designs, the CAR does not bind tumor epitopes directly. Target recognition is conferred by exchangeable adaptor molecules, allowing plug-and-play retargeting and a pharmacologic on/off switch by withholding the adaptor [140]. In the modular universal CAR (UniCAR)-NK format, the receptor recognizes the universal peptide E5B9. Tumor specificity is provided by replaceable target modules (TMs) that carry E5B9 on one end and a tumor-binding moiety on the other. Swapping or combining TMs enables antigen switching and dose titration, addressing heterogeneity while adding drug-like control and safety [141,142]. Adapter CAR (AdCAR)-NK utilizes biotin-based affinity pairs to engage biotinylated antibodies against diverse antigens, supporting an off-the-shelf and tunable targeting system [143,144]. Convertible-CAR, developed in T cells, employs an orthogonal NKG2D ectodomain that binds only to a matched MicAbody adaptor bearing the orthogonal ligand and any desired scFv. Adjusting adaptor identity and dose enables on-demand target switching and graded activation in preclinical studies [145]. Together, these modular schemes couple broad antigen coverage with reversibility and switchability, a desirable profile for solid tumors characterized by dynamic evolution and spatial heterogeneity.

Rigorous target selection is central to CAR design, since truly tumor-specific antigens minimize the risk of on-target off-tumor toxicity [146]. An ideal solid tumor CAR target is highly and homogeneously expressed across malignant cells, shows low or negligible expression in essential normal tissues, and is biologically associated with oncogenic fitness to limit antigen loss [146]. Functional validation is equally critical, encompassing surface accessibility and stability, epitope density and internalization kinetics, spatial distribution across primary and metastatic sites, and relationships with immune evasion pathways [146]. In line with these principles, CAR-NK programs entering clinical evaluation have tended to focus on targets that are abundant and comparatively homogeneous in tumors with minimal normal tissue expression and mechanistic ties to tumor maintenance. Representative trials and key design features are summarized in Table 1.

**Table 1.** Key target antigens of CAR-NK therapy in solid tumors

**Target antigen**	**Indication**	**Product**	Phase	Study objectives	NCT ID
5T4 (TPBG)	Solid tumors	Anti-5T4 CAR-NK cells	Early phase 1	Evaluate safety, tolerability, PK, and initial efficacy	NCT05194709
5T4 (TPBG)	Solid tumors	Anti-5T4 CAR-raNK cells	Early phase 1	Evaluate safety, tolerability, PK, and initial efficacy	NCT05137275
ROBO1	Solid tumors	ROBO1 CAR-NK cells	Phase I/II	Evaluate safety and effectiveness	NCT03940820
ROBO1	Pancreatic cancer	BiCAR-NK (ROBO1 CAR-NK)	Phase I/II	Evaluate safety and efficacy	NCT03941457
CLDN6/GPC3/MSLN/AXL	Ovarian cancer, testis cancer, endometrial cancer	Claudin-6, GPC3, MSLN, or AXL CAR-NK cells (expression-selected)	Phase I	Evaluate safety and preliminary efficacy	NCT05410717
MSLN	Epithelial ovarian cancer	Anti-MSLN CAR-NK cells	Early phase 1	Evaluate safety and efficacy	NCT03692637
TROP2	Solid tumors	TROP2-CAR/IL-15-transduced CB-NK cells	Phase I	Dose-finding; recommend dose	NCT06066424
TROP2	Pancreatic cancer, ovarian cancer, adenocarcinoma	TROP2-CAR/IL-15-transduced CB-NK cells	Phase I/II	Dose-finding; recommend dose	NCT05922930
TROP2	Non-small cell lung cancer	Anti-TROP2 CAR-NK cells	Phase I/II	Evaluate safety and efficacy	NCT06454890
TROP2	Colorectal cancer (minimal residual disease)	TROP2-CAR/IL-15-transduced CB-NK cells	Phase I	Determine safety, tolerability, MTD and RP2D	NCT06358430
CD70	Renal cell carcinoma, mesothelioma, osteosarcoma	CAR.70/IL-15-transduced CB-derived NK cells	Phase I/II	Dose-finding; recommend dose	NCT05703854
NKG2DL	Solid tumors	NKG2D CAR-NK cells	Phase I	Evaluate safety and feasibility	NCT03415100
NKG2DL	Solid tumors (arterial infusion strategy)	NKG2D CAR-NK ± CAR-T	Phase I	Evaluate safety and preliminary efficacy	NCT07021534
NKG2DL	Colorectal cancer	NKG2D CAR-NK cells	Phase I	Evaluate safety and feasibility	NCT05213195
NKG2DL	Pancreatic cancer	NKG2D CAR-NK cells	Early phase 1	Evaluate safety and antitumor activity	NCT06503497
NKG2DL	Pancreatic cancer	NKG2D CAR-NK cells	Early phase 1	Evaluate safety and antitumor activity	NCT06478459
NKG2DL	Ovarian cancer	NKG2D CAR-NK cells	Not applicable	Explore MTD; observe safety and activity	NCT05776355
NKG2DL	Solid tumors	NKG2D CAR-NK cells	Phase I	Evaluate safety and effects	NCT05528341
MICA/B	Non-small cell lung cancer, colorectal cancer, breast cancer, ovarian cancer, pancreatic cancer, head and neck cancer, gastroesophageal cancer	MICA/B CAR-NK(FT536)	Phase I	Determine RP2D	NCT05395052
DLL3	Small cell lung cancer	DLL3 CAR-NK cells	Phase I	Evaluate safety and efficacy	NCT05507593
Claudin 18.2	Gastric cancer, pancreatic cancer	Claudin 18.2 CAR-NK cells	Phase I	Determine MTD and dose-limiting toxicity	NCT06464965
GPC3	Hepatocellular carcinoma	GPC3 CAR-NK (SN301A)	Early phase 1	Evaluate safety, tolerability, and anti-cancer activity	NCT06652243
HER2	Glioblastoma	NK92/5.28.z (HER2 CAR-NK92)	Phase I	Evaluate safety/tolerability; determine MTD or MFD	NCT03383978
HER2	Breast cancer, gastric cancer	HER2 CAR-NK (AB-201)	Phase I/II	Evaluate safety and tolerability	NCT05678205
MUC1	Hepatocellular carcinoma, non-small cell lung cancer, pancreatic cancer, triple-negative invasive breast cancer, malignant glioma of brain, colorectal cancer, gastric cancer	Anti-MUC1 CAR-pNK cells	Phase I/II	Evaluate safety and effectiveness	NCT02839954
PD-L1	Solid tumors	PD-L1 targeting high-affinity NK (t-haNK) cells	Phase I	Evaluate safety, preliminary efficacy; determine MTD	NCT04050709
PD-L1	Pancreatic cancer	PD-L1 t-haNK cells	Phase II	Compare efficacy and overall safety	NCT04390399
PD-L1	Triple-negative breast cancer	PD-L1 t-haNK cells	Phase I/II	Evaluate safety and efficacy	NCT04927884
PD-L1	Gastric cancer, gastroesophageal junction adenocarcinoma, head and neck squamous cell carcinoma	PD-L1 t-haNK cells	Phase II	Evaluate effectiveness	NCT04847466
PD-L1	Glioblastoma	PD-L1 t-haNK cells	Phase II	Evaluate safety and efficacy	NCT06061809
PD-L1	Head and neck squamous cell carcinoma	PD-L1 t-haNK cells	Phase II	Evaluate pathologic tumor response (pTR)	NCT06161545
PD-L1	Head and neck squamous cell carcinoma	PD-L1 t-haNK cells	Phase II	Test safety and efficacy	NCT06239220
PSMA	Prostate cancer	PSMA CAR-NK (TABP EIC)	Early phase 1	Evaluate safety, tolerability, and preliminary efficacy	NCT03692663

5T4, trophoblast glycoprotein 5T4; AXL, AXL receptor tyrosine kinase; BiCAR, bispecific chimeric antigen receptor; CAR, chimeric antigen receptor; CB, cord blood; CB-NK, cord blood-derived NK cell; CLDN18.2, claudin 18.2; CLDN6, claudin-6; DLL3, Delta-like ligand 3; GPC3, glypican-3; HER2, human epidermal growth factor receptor 2; MFD, maximum feasible dose; MICA/B, MHC class I chain-related protein A/B; MSLN, mesothelin; MTD, maximum tolerated dose; MUC1, mucin-1; NK, natural killer; NKG2DL, NKG2D ligands; NCT, ClinicalTrials.gov identifier; PD-L1, programmed death-ligand-1; PK, pharmacokinetics; pNK, peripheral blood-derived NK cell; PSMA, prostate-specific membrane antigen; pTR, pathologic tumor response; ROBO1, roundabout guidance receptor 1; RP2D, recommended phase 2 dose; t-haNK, high-affinity NK variant; TPBG, trophoblast glycoprotein; TROP2, trophoblast cell-surface antigen-2

### Enhancing the persistence of CAR-NK cells

#### Cell-intrinsic cytokine sufficiency

Armored CAR-NK designs aim to supply cytokine support in cis rather than via systemic infusion (Fig. 2D). In a phase I study from MD Anderson, anti-CD19 CAR-NK cells encoding IL-15 and an inducible caspase 9 (iCasp9) safety switch persisted for months to a year without exogenous cytokines and achieved high early response rates with a favorable safety profile, supporting the feasibility of IL-15-armored CAR-NK cells to achieve prolonged persistence in humans [7]. IL-15-secreting CD70 CAR-NK cells have likewise been shown in xenograft models to enhance in vivo persistence and antitumor activity [147]. Embedding IL-15 within the CAR architecture of CAR-NK cells and deletion of the cytokine checkpoint CISH preserved the signal transducer and activator of transcription 5 (STAT5)–mTORC1–MYC axis and a balanced glycolysis–oxidative phosphorylation program, which was associated with prolonged detectability and improved tumor control [148].

In parallel with IL-15, ex vivo expansion platforms delivering membrane-bound IL-21 (mbIL-21) in cis can delay telomere attrition, limit senescence, and sustain the proliferative capacity of NK cells, translating into improved persistence and antitumor activity [149,150]. Consequently, the K562–mbIL-21–4-1BBL artificial antigen-presenting cell (APC) system has become one of the most widely used platforms for clinical-grade NK expansion and can be readily adapted for CAR-NK manufacturing.

For clinical exogenous support, the IL-15 superagonist complex N-803 (ALT-803) mimics physiologic IL-15/IL-15Rα trans-presentation and drives robust expansion and activation of NK and CD8^+^ T cells in vivo with an acceptable safety profile in early-phase trials [151,152]. On this basis, N-803 is being explored as a cytokine adjuvant for CAR-NK therapies, although data are still emerging.

#### Cytokine-induced memory-like phenotype

Brief priming with IL-12, IL-15, and IL-18 for 12 to 16 h reprogrammed NK cells into a cytokine-induced memory-like (CIML) state [153,154]. Upon restimulation, these cells displayed augmented IFN-γ production and degranulation, and they maintained prolonged in vivo detectability with more stable function [153,154]. A first-in-human phase I study in AML established safety and in vivo expansion with an overall response rate of 55% [153]. Superimposing CAR design on CIML NK cells boosted antigen-specific responses and recall capacity without compromising specificity [155–157]. In NK-resistant lymphoma models, CAR-modified CIML NK cells (CAR-ML NK) showed increased IFN-γ production, greater degranulation, and superior tumor suppression in vivo. Survival was substantially prolonged in human xenografts, indicating additive benefits of CIML programming and CAR signaling with concurrent gains in effector function and persistence [155]. Epitope-refined CAR-ML NK cells achieved deeper disease control and longer persistence in *NPM1*-mutant AML [156]. In solid tumor settings, CAR-ML NK cells targeting the proximal membrane domain of MSLN produced marked antitumor and anti-metastatic activity in ovarian cancer models, underscoring their plasticity and resilience within the TME [157].

#### Anti-apoptotic engineering and metabolic adaptation

Gene-editing strategies that delete pro-apoptotic regulators or enforce expression of anti-apoptotic BCL-2 family members (BCL-2, BCL-xL, MCL-1) could buffer TME-driven death cues and improve persistence [158]. These interventions carry safety liabilities because the same pathways support tumor immune evasion. Expression of anti-apoptotic genes should therefore be time-limited and titratable, ideally coupled to druggable safety switches. At the signaling level, the cytokine-inducible SH2-containing protein CISH functions as a negative regulator of the IL-15–JAK–STAT axis in NK cells [159]. CISH disruption in IL-15-armored CAR-NK cells enhanced the Akt/mTORC1/c-MYC cascade and glycolytic flux, improved metabolic “fitness”, reduced stress-induced apoptosis, and markedly prolonged persistence of CAR-NK cells in vivo [148,159]. Relief of extrinsic death and exhaustion programs can convert functional durability into survival durability. A2A/A2B receptor antagonists and dominant-negative TGF-β receptors blunted TME-induced apoptosis and exhaustion [160,161]. Antioxidant engineering provides a complementary route. Enhancing resistance to ROS or equipping NK cells with antioxidant enzymes mitigated metabolic suppression under oxidative stress and strengthened antitumor activity, providing a rationale for analogous antioxidant “armoring” in future CAR-NK designs [162].

#### Checkpoint blockade and niche conditioning

Blocking inhibitory checkpoints such as NKG2A, TIGIT, and PD-1 helps sustain NK cell activity in nutrient-poor, suppressive TMEs and may indirectly prolong in vivo persistence [163]. In preclinical NK models, anti-TIGIT monoclonal antibodies (mAbs) restored function in exhausted NK cells and extended intratumoral activity [163]. CAR-NK cells derived from allogeneic hematopoietic stem and progenitor cells (HSPCs) or induced pluripotent stem cells (iPSCs) exhibited greater longevity and self-renewal potential, offering a route to improved persistence [164]. Another complementary approach is lymphodepleting preconditioning before adoptive CAR-NK transfer to establish a permissive niche for expansion. By depleting endogenous lymphocyte populations that compete for space and cytokines and by attenuating host immune rejection, these regimens allow the infused CAR-NK cells to expand more freely [165]. The cyclophosphamide plus fludarabine regimen is commonly used to support allogeneic CAR-NK therapy, although optimal intensity and combinations are still being refined [165].

### Engineering safety into CAR-NK therapy

Enhancing the safety of CAR-NK therapy often involves co-expression of an inducible suicide switch (Fig. 2E). The iCasp9 module functions as a failsafe, where CAR-NK cells are engineered to express a chimeric caspase-9 fused to a drug binding domain. Administration of the dimerizer rimiducid (AP1903) induces homodimerization and triggers apoptosis, enabling rapid pharmacologic ablation of the infused cells [166]. This design has been incorporated into a cord blood-derived CD19-CAR/IL-15/iCasp9-NK product and showed a favorable safety profile, with no switch activation required during the study, supporting feasibility and controllability [7]. Concordant preclinical and early clinical data indicate that iCasp9-based safety switches may help mitigate potential toxicities while preserving antitumor activity [7,167]. Additional safety layers use logic gating and locoregional delivery to concentrate activity within the tumor and limit systemic exposure. AND gates require concurrent recognition of 2 antigens to reach the activation threshold. Coupling an AND gate with a NOT gate yields AND-NOT logic that excludes cells expressing a normal lineage marker despite single-antigen positivity, thereby improving tissue selectivity [168].

## Synergistic Combinations of CAR-NK Cells with Other Therapies

**Fig. 3.**
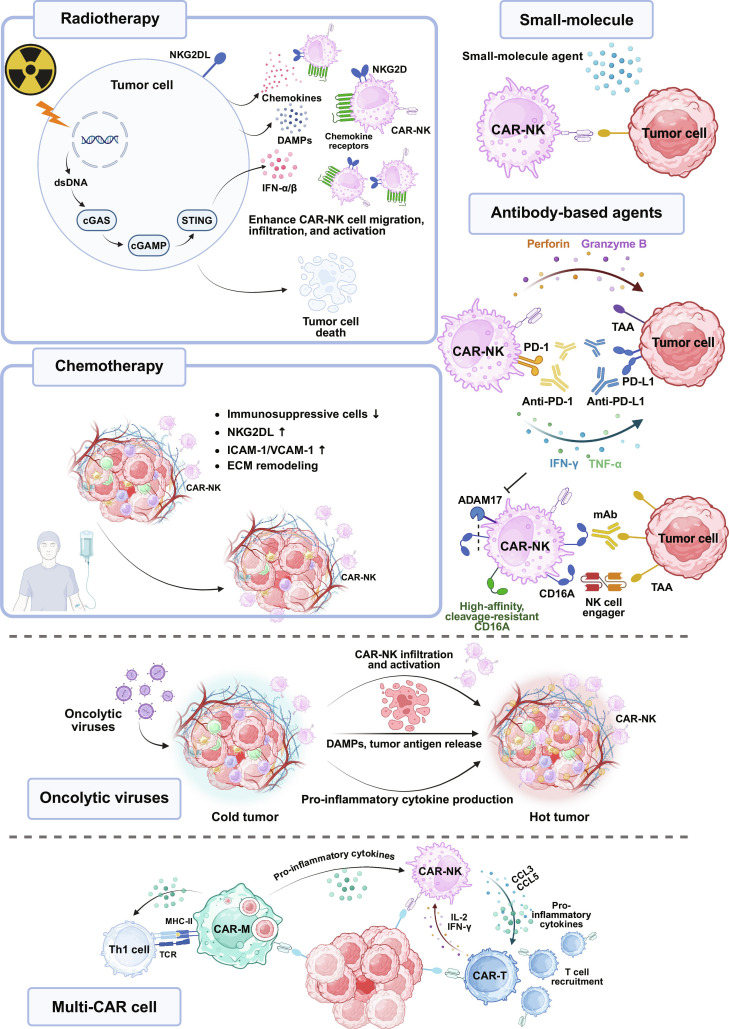
Combination strategies to potentiate CAR-NK therapy in solid tumors. Radiotherapy, chemotherapy, small molecules, antibody-based agents, oncolytic viruses, and multi-CAR cell regimens work together to remodel the tumor microenvironment and heighten tumor visibility. These approaches strengthen chemokine signaling and vascular access, ease extracellular matrix barriers, and increase danger signals and antigen exposure. Figures were created with BioRender.com. CAR, chimeric antigen receptor; CAR-M, chimeric antigen receptor macrophage; CCL, C–C motif chemokine ligand; DAMPs, damage-associated molecular patterns; ECM, extracellular matrix; ICAM-1, intercellular adhesion molecule-1; IFN-γ, interferon-γ; mAb, monoclonal antibody; MHC-II, major histocompatibility complex class II; NKG2DL, NKG2D ligand; TAA, tumor-associated antigen; TCR, T cell receptor; TNF-α, tumor necrosis factor-α; VCAM-1, vascular cell adhesion molecule-1.

Key combination approaches integrating CAR-NK cells with RT, chemotherapy, immune checkpoint blockade, and locoregional delivery are schematically summarized in Fig. 3.

### Chemotherapy

Rationally designed chemotherapy can do more than tumor reduction; it can help establish an entry- and expansion-permissive niche for adoptively transferred lymphocytes, including CAR-NK cells [85,169,170]. Lymphodepletion with cyclophosphamide and fludarabine removes endogenous competitors, increases availability of homeostatic cytokines such as IL-7 and IL-15, and thereby supports in vivo expansion of infused effector cells [85,171]. Anthracyclines, platinum agents, and etoposide up-regulate NKG2D ligands and death receptors and induce immunogenic cell death (ICD), a form of tumor cell death that releases danger signals and promotes antigen presentation and T cell priming, thereby potentially increasing tumor susceptibility to CAR-NK cytotoxicity and T cell cross-priming [172–174]. Taxanes and platinum agents have also been reported to remodel tumor stroma, up-regulate ICAM-1 on endothelial cells, and improve perfusion, changes that could lower barriers to extravasation and deep parenchymal access for CAR-NK cells [175,176]. Low-dose or metronomic 5-fluorouracil can preferentially suppress MDSCs while relatively sparing effector compartments, providing a rationale to combine such regimens with CAR-NK therapy to relieve myeloid suppression [177].

In preclinical models, cisplatin priming sensitized ovarian tumors and enabled subsequent CAR-NK therapy to enhance killing of cancer stem cell populations [178,179]. Furthermore, drug-loaded CAR-NK cells (e.g., surface-anchored paclitaxel liposomes) delivered chemotherapy directly to tumor sites, achieving high local exposure with controlled systemic toxicity [180,181]. This approach improved tumor control both in vitro and in vivo and increased intratumoral drug concentrations without injuring the carrier CAR-NK cells [180,181]. Clinically, several early-phase trials are exploring CAR-NK infusions in combination with chemotherapy in pancreatic cancer and relapsed/refractory NSCLC (NCT06503497, NCT06454890), but optimal dosing, scheduling, and sequencing remain to be defined. Evidence-based optimization of dose, cycle, and combination partners remains essential to balance tumor burden reduction with preservation of immune competence while minimizing adverse effects.

### Radiotherapy

Combining RT with CAR-NK therapy is emerging as a promising strategy to overcome barriers in solid tumors. Rational RT can do more than local cytotoxicity, functioning as a “systemic primer” for CAR-NK trafficking and activity. Ionizing radiation induces tumor cell DNA damage and activates the cGAS-STING/type I IFN axis, thereby increasing CXCL9/CXCL10 expression and amplifying danger-associated molecular pattern (DAMP) signals—stress cues released by damaged tumor cells that recruit and activate immune cells [182–184]. RT also up-regulates endothelial ICAM-1/VCAM-1, promotes transient vascular normalization, and reduces hypoxia [184,185]. Together, these changes lower the physical threshold for transendothelial migration and deep parenchymal infiltration, thereby enhancing CAR-NK homing and intratumoral accumulation [184–186]. RT further elevates proinflammatory cytokines including IFN-γ and TNF-α, supporting CAR-NK activation and persistence [187,188]. In addition, radiation-induced DNA damage can up-regulate NKG2D ligands on tumor cells, heightening NK cell activation and cytotoxicity, a pathway that could be leveraged by NKG2D-based CAR-NK designs [189,190]. However, direct clinical evidence for these RT-CAR-NK synergies is still lacking and will require carefully designed combination trials.

Preclinical studies support synergy between RT and CAR-NK cell therapy. In HCC, GPC3-CAR-NK combined with RT demonstrated stronger antitumor activity in vivo [114]. Robo1 CAR-NK92 with RT likewise enhanced NK cell trafficking, prolonged survival in solid tumor mouse models, and improved tumor control [128]. However, high-dose irradiation may drive excessive immunosuppressive remodeling and blunt CAR-NK activity. Lower fractional doses are more likely to elicit immunogenic responses. Future research should systematically optimize dose, fractionation, and sequencing, and carefully integrate additional immunomodulators to maximize therapeutic benefit.

### Antibody-based agents

Antibody-based agents can support CAR-NK therapy through 3 complementary routes. First, ICIs relieve NK dysfunction and may augment CAR-NK responses, as suggested by preclinical studies. Second, tumor-targeting mAbs engage CD16A to drive ADCC and amplify killing. Third, antibody–drug conjugates (ADCs) and NK cell engagers (NKCEs) such as bispecific NK cell engagers/trispecific NK cell engagers (BiKEs/TriKEs) broaden antigen coverage and enhance immunogenicity within the TME.

#### Immune checkpoint inhibitors

Inhibitory checkpoints such as PD-1 are critical regulators of lymphocyte activation [191]. For NK cells, however, PD-1 expression itself remains controversial [192–194]. Early studies described PD-1 up-regulation on tumor-infiltrating NK cells in several malignancies and associated PD-1^+^ NK subsets with impaired degranulation, functional exhaustion, and poor outcomes, with PD-1/PD-L1 blockade partially restoring NK cell function [192,193]. In contrast, a systematic analysis using stringent gating, multiple PD-1 antibody clones, and transcriptomic profiling found that mouse and human NK cells express little to no PD-1 at baseline or after activation [194], suggesting that PD-1 is not a universal NK checkpoint and that earlier findings may be influenced by technical factors and context-dependent expression. Moreover, overexpressing PD-L1 on allogeneic CAR-NK cells engaged PD-1 on recipient T cells, dampened allorejection, and augmented antitumor activity [195]. Taken together with the expression data above, these findings indicate that, although the extent of NK-intrinsic PD-1 expression is debated, the PD-1/PD-L1 pathway remains a relevant lever to modulate the microenvironment for CAR-NK therapy.

In a humanized nasopharyngeal carcinoma model, Liu et al. [196] showed that PD-L1-targeted CAR-NK cells plus the anti-PD-1 antibody (nivolumab) produced synergistic tumor suppression that outperformed either monotherapy. Anti-PD-L1 markedly boosted the antitumor efficacy of CLDN6-CAR-NK cells in ovarian cancer models [197]. Additional work indicates that pairing CAR-NK cells with PD-1 blockade can recondition an immunosuppressive TME, increase intratumoral T cell and NKT cell infiltration, and control tumors that are refractory to CAR-NK alone [198].

Early clinical experience is emerging. In recurrent HER2-positive glioblastoma, the phase I (CAR2BRAIN) cohort testing local administration of HER2-CAR-NK92/5.28.z demonstrated feasibility and a favorable safety profile [199]. Under the same registration, a combination cohort with the PD-1 inhibitor (ezabenlimab) is ongoing (NCT03383978), with full clinical results yet to be published. Another phase II clinical trial is evaluating PD-L1 CAR-NK combined with pembrolizumab and the IL-15 superagonist N-803 in recurrent/metastatic gastric or HNC (NCT04847466). Beyond the PD-1/PD-L1 pathway, other inhibitory receptors are also being targeted in combination with CAR-NK cells. Recent preclinical studies suggested that disrupting TIGIT signaling in NK or CAR-NK cells can relieve inhibitory cues and enhance control of TIGIT ligand-enriched solid tumor models [200]. Inhibitory receptors such as LAG-3 and TIM-3 similarly constrain NK cell effector function, and blockade of these pathways is being explored to further potentiate CAR-NK activity [201,202].

#### Monoclonal antibodies

mAbs can, in principle, synergize with CAR-NK cells through CD16A-mediated ADCC, thereby amplifying antitumor activity. Early clinical experience with NK cell therapies has suggested improvements in overall survival or objective responses in certain settings [203]. Trials combining mAbs with CAR-NK are ongoing in advanced solid tumors, including cetuximab (anti-EGFR, NCT06239220) for HNC and bevacizumab (anti-VEGF-A, NCT06061809) for glioblastoma. Cytokine activation or target cell engagement may trigger ADAM17-mediated shedding of CD16 on NK cells, which blunts ADCC [204]. Engineering noncleavable or high-affinity CD16 variants and providing autocrine IL-15, exemplified by the iPSC-derived CD19 CAR-NK product FT596, is designed to stabilize ADCC and has demonstrated antitumor activity with good tolerability in a phase I study (NCT04245722) [205].

#### ADCs and NKCEs

ADCs achieve selective payload delivery and can also induce ICD and bystander effects, thereby promoting antigen spread in ways that may complement adoptive cell therapy [206,207]. Leveraging retargetable adaptor design, universal platforms such as SNAP-CAR enable on-demand switching with benzylguanine (BG)-labeled antibody or ADC adaptors, allowing rapid redirection of specificity and suggesting a framework to “ADC plus CAR” complementarity [208,209]. These studies have so far been conducted primarily in CAR-T systems, and whether such designs can translate into clinically meaningful synergy with CAR-NK cells remains to be established. NKCEs, including BiKEs and TriKEs, potentiate ADCC effects by co-engaging CD16 and tumor antigens, and IL-15-containing TriKEs deliver an intrinsic cytokine signal that supports NK cell survival, proliferation, and function under cytokine-poor settings [210–212]. Early phase studies with TriKEs GTB-3550 have reported favorable safety together with endogenous NK expansion and activation [211]. These data provide a rationale for testing NKCEs as adjuncts to CAR-NK therapy to enhance persistence and bystander immunity, although such combination strategies have yet to be systematically evaluated.

### Small-molecule agents

Multi-targeted TKIs and other targeted small molecules can complement CAR-NK therapy by increasing tumor susceptibility to CAR-NK cytotoxicity and curbing malignant proliferation [213–216]. Anti-angiogenic TKIs such as sorafenib and axitinib can transiently normalize tumor vasculature and dampen VEGF-driven signaling, which alleviates immune suppression within the TME and can improve effector cell infiltration [217]. Sunitinib, widely used for renal cell carcinoma, reduced peripheral and intratumoral MDSCs and Tregs, creating a milieu more permissive for antitumor immune activation [218]. Consistent with this, regorafenib combined with EpCAM-CAR-NK cells demonstrated superior tumor control in CRC models, and cabozantinib up-regulated EGFR while down-regulating PD-L1, markedly enhancing the cytotoxicity of EGFR-CAR-NK cells in renal cancer [213,214]. Additional small molecules such as IDO inhibitors, adenosine A2A receptor antagonists, CSF1R blockers, histone deacetylase (HDAC) inhibitors, and STING agonists can modulate metabolism and myeloid compartments, offering opportunities for combination with CAR-NK therapy [219–221]. Proteasome inhibitor pretreatment with bortezomib amplified CAR-NK effects by remodeling leukemic surface immunophenotypes in a bidirectional manner, thereby reducing disease burden and prolonging survival in AML models [215]. The HDAC inhibitor entinostat maintained and augmented CAR expression in vitro and in vivo, improving persistence and antitumor activity of CAR-NK cells [216]. Taken together, preclinical data support small-molecule and CAR-NK combinations, while resistance evolution, off-target toxicities, short half-lives, and limited penetration in solid tumors remain key obstacles to clinical translation [221]. Greater molecular selectivity and improved delivery, including local or controlled-release formats, along with rational combinations and optimized sequencing, will be required to achieve durable and clinically meaningful benefit.

### Oncolytic viruses

Oncolytic viruses (OVs) are emerging cancer therapeutics that preferentially replicate in tumors and induce ICD, releasing DAMPs and tumor antigens. These events can activate cellular stress programs, up-regulate adhesion molecules, and rewire chemokine circuits, thereby helping convert cold tumors into hot lesions [222]. In glioblastoma models, IL-15/IL-15Rα-armed OV (OV-IL15C) combined with EGFR-CAR-NK cells suppressed tumor growth and prolonged survival, accompanied by increased intracranial infiltration and activation of NK cells and CD8^+^ T cells and improved CAR-NK persistence in immunocompetent settings [223]. Similar synergy has been observed in neuroblastoma and pancreatic cancer models, where cytokine-secreting OVs combined with CAR-NK cells deliver superior antitumor activity [224,225]. In addition, CAR-NK cells targeting avsialidase (a membrane-bound sialidase) in combination with an avsialidase-expressing oncolytic vaccinia virus is being developed as a strategy that may improve efficacy and broaden target coverage in solid tumors [226].

### Multi-CAR cell combinations

CAR-T, CAR-NK, and CAR-macrophage (CAR-M) each possess distinct strengths, and their integration may help offset platform-specific limitations in solid tumors [9,227–229]. CAR-T cells deliver exceptional antigen specificity and persistence, yet exhibit restricted homing and penetration and are susceptible to functional exhaustion within suppressive TMEs [9]. In contrast, CAR-NK cells provide innate cytotoxicity and CD16A-mediated ADCC, which may help clear antigen-low or antigen-loss variants that escape CAR-T recognition [9,227]. CAR-M remodel the TME through phagocytosis, antigen presentation, and pro-inflammatory reprogramming, changes that could help prime and sustain CAR-T and CAR-NK activity in situ [228,229].

#### CAR-T

Combined CAR-T and CAR-NK therapy may offer complementary advantages over either platform alone in solid tumors [13]. CAR-NK cells can recruit and enhance CAR-T intratumoral trafficking by secreting chemokines such as CCL3, CCL5, and IL-8, while activated CAR-T cells in turn enhance NK cell activation and persistence by releasing cytokines including IL-2 and IFN-γ [13]. This bidirectional amplification may reduce relapse risk, but this remains to be validated clinically. Co-administration of CD19-targeted CAR-NK and CAR-T cells has shown rapid and durable tumor control while lowering pro-inflammatory cytokine release in xenograft models [230].

#### CAR-M

The combination of CAR-NK with CAR-M could, in principle, exploit an inflammation-driven remodeling and antigen presentation strategy that both primes and consolidates antitumor responses. CAR-M traffics into hypoxic and necrotic niches, rewires the TME to support antitumor immune responses, secretes IL-12 and TNF-α, and functions as an APC to broaden antigen spread [231–233]. In a phase I study, anti-HER2 CAR-M (CT-0508) was safe, remodeled the intratumoral milieu, and expanded CD8^+^ T cells, suggesting that CAR-M could serve as a priming and companion modality that improves infiltration and response quality [234].

Given these complementary strengths, unifying CAR-T, CAR-NK, and CAR-M within a single treatment paradigm may leverage orthogonal mechanisms to better address key obstacles in solid tumors. Such combinations warrant evaluation in early-phase trials to determine whether they can deepen and sustain immune responses and ultimately improve patient outcomes.

### Cytokines

Cytokines delivered systemically or encoded as “armored” autocrine payloads can increase CAR-NK expansion, persistence, and cytotoxicity. IL-2 can transiently sustain or boost CAR-NK effector activity, yet systemic administration provokes vascular leak and Treg expansion, substantially limiting in vivo utility [235,236]. In contrast, IL-15, including the superagonist N-803, preferentially expands and activates NK and CD8^+^ T cells with less Treg stimulation, making it an attractive candidate for priming and maintenance regimens around CAR-NK infusion [152,237]. However, correlative analyses from haploidentical NK cell trials have shown that systemic N-803 support reduced NK cell persistence and clinical activity compared with IL-2, likely by driving expansion of recipient CD8^+^ T cells and accelerating rejection of donor NK cells [238]. These findings suggest that systemic IL-15 superagonism may require careful recipient-immune management in allogeneic CAR-NK programs and motivate strategies that spatially or cell-restrictedly deliver IL-15 signaling. Membrane-bound IL-15 and IL-15/IL-15R fusion formats, which confine NK cell activation to zones of cell contact and can help limit bystander activation in preclinical studies [239], have been incorporated into several iPSC-derived CAR-NK products, including Fate Therapeutics’ FT576 and FT522 to reduce systemic cytokine exposure. Furthermore, calibrated-release IL-15 design uses a protease-cleavable membrane anchor to enable local short-range release, as in SENTI-202 (NCT06325748) and SN301A (NCT06652243). For solid tumors, local or conditional cytokine delivery may amplify intratumoral immune responses while minimizing systemic toxicity, making it particularly attractive for high-risk lesions.

## Future Directions: Expanding the Potential of CAR-NK Cells in Solid Tumors

### Next-generation target landscapes and multi-antigen logic for CAR-NK

A central priority for the next wave of CAR-NK development in solid tumors is to move beyond a small set of conventional antigens and systematically redefine the target landscape. Because solid tumors display marked intra- and interpatient heterogeneity and many candidate antigens show low-level expression in normal tissues, future targets must be selected under competing constraints of coverage and safety [13]. Recent advances in single-cell and spatial multi-omics now allow high-resolution mapping of tumor, stromal and immune compartments, and the identification of tumor-restricted surface antigens, while integrated transcriptomic and proteomic datasets help quantify heterogeneity and on-target, off-tumor risk [163,240].

Future target discovery for CAR-NK should therefore evolve from simply listing TAAs to implementing standardized pipelines that (a) nominate candidates using multi-omics data, (b) functionally validate them in engineered models, (c) screen normal tissue atlases for safety, and (d) incorporate manufacturability and population coverage early in development [13,240,241]. In parallel, multi-antigen logic will likely become a defining feature of next-generation CAR-NK designs. Tandem/OR-gate CARs may help address antigen heterogeneity, while AND- or NOT-gate designs and combinations of tumor-restricted antigens with stress ligands (e.g., NKG2D ligands) could improve discrimination and limit antigen-loss escape [163]. However, most multi-antigen CAR-NK constructs remain at an early preclinical stage, and it is unclear whether added complexity yields meaningful benefit or merely increases manufacturing and regulatory burden; future studies must identify which antigen logics truly improve depth and durability of response in vivo.

### Reprogramming CAR-NK metabolism and mitochondrial fitness

Even when CAR-NK cells successfully traffic to tumors, the hypoxic, nutrient-depleted, and metabolite-rich microenvironment rapidly erodes their effector function [242,243]. Recent work has highlighted that hypoxia, lactate accumulation, and arginine depletion drive mitochondrial fragmentation, impaired oxidative phosphorylation, and diminished cytotoxicity in tumor-infiltrating NK cells [242,243]. In preclinical models, hypoxia-induced activation of the HIF-1α–mTOR–DRP1 axis promoted excessive mitochondrial fission and NK cell exhaustion, whereas disrupting DRP1 can partially rescue function under low oxygen [61,243]. These studies collectively argue that mitochondrial dynamics and metabolic flexibility are not epiphenomena but central determinants of NK cell persistence in solid tumors.

For CAR-NK therapy, this points toward a new class of engineering strategies that complement receptor signaling with metabolic reprogramming. Conceptually, CAR-NK cells could be endowed with enhanced mitochondrial biogenesis, more efficient mitophagy to clear damaged organelles, or altered expression of nutrient transporters and key metabolic enzymes to better tolerate glucose and arginine scarcity [244–246]. In parallel, small molecules targeting lactate export, adenosine signaling, or other metabolic checkpoints could be combined with CAR-NK to create a more permissive bioenergetic niche [247,248]. Such strategies may synergize with cytokine-based persistence modules discussed above but will require dedicated safety frameworks to avoid unintended long-term consequences. To date, however, most evidence for such approaches comes from non-engineered NK cells or from T cell models, and there is minimal in vivo data for metabolically rewired CAR-NK products. The long-term safety of “metabolically boosted” NK cells is also uncertain, as sustained high metabolic activity may accelerate exhaustion or even increase genomic instability.

A priority for future work is therefore to test metabolically engineered CAR-NK cells in TME-mimicking systems, including hypoxic, nutrient-poor 3-dimensional (3D) cultures and organoid cocultures, with longitudinal profiling of mitochondrial function and exhaustion markers. Only by coupling mechanistic insight with rigorous safety evaluation can metabolic rewiring become a credible and translatable axis of CAR-NK optimization instead of a one-off proof-of-concept in select models.

### Systemic modulators of CAR-NK function: Microbiome, host metabolism, and beyond

Most current CAR-NK development focuses on tumor- or cell-intrinsic barriers, yet accumulating evidence indicates that systemic factors—particularly the gut microbiota and host metabolic state—can shape antitumor immunity. A rapidly expanding body of work has shown that the gut microbiota can substantially influence responses to ICIs and CAR-T cell therapy, in part by remodeling systemic cytokine milieus and immune cell differentiation [249,250]. Short-chain fatty acids, secondary bile acids, and tryptophan metabolites produced by gut bacteria can reprogram NK cell metabolism and epigenetic states, thereby tuning their maturation, cytokine production, and cytotoxicity in peripheral and tumor sites [251,252]. These data suggest that the therapeutic window of CAR-NK cells may also depend on a “microbiome context” that has so far been largely ignored in trial design. However, CAR-NK-specific data remain sparse, and current inferences largely extrapolate from broader immunotherapy settings.

In the future, integrating microbiome and host metabolic profiling into CAR-NK trials will be important to move from correlation to causality. Prospective collection of stool, blood, and tumor samples could allow association of baseline microbial signatures, metabolomic patterns, or nutritional states with CAR-NK expansion, persistence, and toxicity [249]. In principle, such datasets may inform rational combination regimens that pair CAR-NK therapy with microbiome-modulating interventions, including probiotics or fecal microbiota transplantation, although this remains to be tested prospectively [253]. Host factors such as obesity, insulin resistance, sarcopenia, and aging are known to reshape the NK compartment, often skewing toward dysfunctional or exhausted phenotypes [254–256]. Rather than treating these as background noise, future studies should systematically examine how such comorbidities influence CAR-NK expansion, trafficking, and toxicities, and whether patient selection or prehabilitation can mitigate adverse effects.

Beyond the microbiome and metabolism, other systemic axes such as neuroendocrine stress responses and circadian rhythms may also modulate NK biology, but their impact on CAR-NK has scarcely been explored. A more holistic view of CAR-NK therapy that integrates local TME factors with systemic modulators may reveal nongenetic levers to improve outcomes. At present, these concepts remain largely hypothetical, and translating them into practice will require carefully designed CAR-NK trials incorporating longitudinal sampling and integrated systems immunology analyses.

### Technology accelerators: AI-guided design, CRISPR screens, and NAM platforms

Several technological trends are poised to accelerate how CAR-NK therapies are discovered, optimized, and de-risked. First, artificial intelligence (AI) and machine learning (ML) can be harnessed at multiple stages of CAR-NK development. On the discovery side, ML models are increasingly used to integrate multi-omic tumor datasets and clinical outcomes, identify immune subtypes, and predict responses to immunotherapies [240]. Applied to CAR-NK, such models could prioritize antigen combinations that maximize tumor coverage while minimizing predicted off-tumor expression or identify patient subsets with NK-enriched immune phenotypes who might benefit most from NK-based products. On the receptor-engineering side, deep learning-based protein design has already been used to refine CAR-T binders; similar approaches may help optimize CAR-NK binding domains, hinge regions, and intracellular signaling modules to balance activation strength, persistence, and exhaustion risk [257,258]. In manufacturing, real-time analysis of expansion kinetics, metabolic parameters, and phenotypic markers could enable predictive models that recommend optimal harvest times and flag failing cultures early, supporting more consistent large-scale production [259].

Second, advances in genome engineering provide powerful tools for “vaccinating” CAR-NK designs against TME-induced dysfunction. CRISPR/Cas9-based perturbation screens in human NK cells are beginning to systematically map genetic circuits that tune cytotoxicity, cytokine responsiveness, and resistance to suppression. A recent genome-wide CRISPR screen of IL-15-stimulated NK cells delineated the IL-15R signaling network and identified ubiquitin-dependent IL-15R degradation as a key brake on NK activation, nominating components such as *UBE2F* and *ARIH2* as druggable targets to boost IL-15-driven antitumor immunity [260]. Biederstädt et al. [261] developed a genome-wide CRISPR platform for primary human NK cells and performed high-content screens under repeated tumor challenge and TME-like stress, uncovering targets including *MED12*, *ARIH2*, and *CCNC* whose deletion markedly enhanced CAR-NK cytotoxicity and persistence. These studies illustrate how pooled CRISPR screens can uncover non-intuitive nodes for engineering next-generation CAR-NK cells with superior fitness in solid tumors.

Third, new approach methodologies (NAMs) offer an opportunity to modernize preclinical evaluation of CAR-NK therapies. Tumor organoids, immune-enhanced organoids, and organ-on-chip systems increasingly recapitulate key aspects of human tumor architecture, stromal composition, and microenvironmental gradients, and are being applied to study interactions between tumors and cellular immunotherapies [262,263]. For CAR-NK, such models can be used to interrogate 3D infiltration patterns, serial killing capacity under hypoxia and nutrient stress, and potential damage to normal organoid counterparts, thereby addressing the central questions of “getting in, functioning, and sparing healthy tissue” in a controlled human-relevant setting. Microfluidic chips with endothelial barriers and in silico NAMs such as toxicity prediction and agent-based simulations may further model trafficking, off-tumor interactions, and dosing strategies [263,264].

Importantly, NAMs are better viewed as complements rather than full replacements for animal models, at least in the near term. Current organoid and chip platforms often lack components such as vasculature, secondary lymphoid structures, and full myeloid repertoires, and their predictive value for clinical CAR-NK outcomes is still being defined [265,266]. Nonetheless, a “NAM-first, animal-refined” pipeline—where CAR-NK constructs are initially screened and stress-tested in advanced in vitro systems, and only the most promising candidates progress to focused in vivo studies—could both accelerate development and improve translatability while aligning with regulatory efforts to reduce animal use without compromising patient safety. For CAR-NK specifically, establishing standardized, NK-adapted NAM platforms and correlating their readouts with early clinical trial data will be critical steps to ensure that these tools are used to answer the most relevant translational questions.

### Clinical translation, manufacturing, and industrial challenges

Despite impressive preclinical progress, the pace at which CAR-NK therapies can move into routine clinical practice will ultimately depend on whether products can be manufactured reproducibly, at scale and at acceptable cost. Current clinical products rely on heterogeneous starting materials, diverse culture platforms, and site-specific protocols, leading to substantial variability in cell phenotype, dose, and potency across trials [165]. Compared with patient-specific CAR-T cells, CAR-NK products are more often conceived as allogeneic, off-the-shelf agents. This creates opportunities for standardized mass production and rapid deployment, but also imposes stringent requirements on sourcing, cryopreservation, and long-term product stability.

A first strategic decision is the choice of starting cell source. Peripheral blood NK cells are attractive because of their accessibility and mature cytotoxic phenotype, but their limited ex vivo expansion potential and relatively low transduction efficiency make large-scale production challenging [267]. Cord blood NK cells exhibit higher proliferative capacity and are more amenable to genetic modification, but each unit contains limited cell numbers and banking logistics are complex [165]. NK92 and related NK cell lines offer a uniform, renewable platform with excellent transducibility, but mandatory irradiation restricts in vivo persistence and typically necessitates repeated dosing [268]. iPSC- or HSPC-derived NK cells promise clonal uniformity, efficient multiplex edits, and virtually unlimited supply, but differentiation protocols remain lengthy, technically demanding, and costly, and only a few have been implemented at full good manufacturing practice (GMP) scale [164,165]. No single platform has emerged as dominant; different indications and health-care settings may ultimately favor different sources, and genuine head-to-head comparisons are still rare [165].

At the process level, CAR-NK manufacturing is transitioning from open, manual flasks to automated, closed systems [269]. Gas-permeable bags, bioreactors, and integrated cell-processing devices can achieve large-scale expansion under GMP, and serum-free, xeno-free media are being adopted to reduce variability and regulatory burden [269]. However, protocols differ widely in starting cell dose, cytokine cocktails, feeder usage, and culture duration. Defining platform-agnostic critical process parameters that correlate with in vivo performance and embedding real-time analytics into process development will be essential to move from artisanal production to robust, transferable manufacturing.

Because most CAR-NK products are envisioned as off-the-shelf agents, cryopreservation and post-thaw performance become central manufacturing challenges [270]. NK cells are unusually sensitive to freeze–thaw stress: Even with acceptable post-thaw viability, degranulation, cytokine secretion, and serial killing can be severely compromised [270,271]. Optimization of cryoprotectant composition, cooling and thawing rates, and post-thaw recovery culture is ongoing, yet no consensus protocol has been established and interlaboratory variability in post-thaw potency remains substantial [270]. Moreover, repeated infusions from the same frozen batch raise unresolved questions about cumulative functional decline and immunogenicity. Future studies should therefore treat cryopreservation, storage, and thawing as integral components of the manufacturing process and systematically link them to clinical pharmacokinetics and outcomes.

Quality control and regulation represent another underdeveloped area. At present, most CAR-NK programs extrapolate release criteria from CAR-T paradigms—such as basic immunophenotyping, viability, and short-term cytotoxicity—despite the distinct biology and effector mechanisms of NK cells [272,273]. There is no consensus on which attributes best predict clinical behavior: Activating/inhibitory receptor balance, exhaustion markers, metabolic fitness, and transcriptional signatures are all candidates but remain weakly validated [273]. Establishing NK-specific critical quality attributes and standardized potency assays will be crucial for regulators to evaluate comparability between lots, sites, and process changes [273]. Data-driven approaches, including AI-assisted analysis of large manufacturing and clinical datasets, may help accelerate this process but will require harmonized reporting and a culture of data sharing that is not yet standard in cell-therapy programs [274].

Finally, cost and global accessibility will determine whether CAR-NK becomes a widely available modality. Heavily engineered, iPSC-derived, or feeder-dependent CAR-NK cells carry substantial cost of goods, and rigorous health-economic analyses are still scarce [275,276]. Global implementation will require not only technological advances—such as modular manufacturing sites, decentralized cryostorage, and simplified infusion protocols—but also innovative business models and reimbursement frameworks that recognize the distinct value proposition of CAR-NK compared with existing therapies [277,278]. In this sense, manufacturing is not only a technical exercise but also a central determinant of which patient populations will ultimately benefit from CAR-NK technology. Overall, the success of CAR-NK therapy in solid tumors will depend not only on overcoming biological barriers but also on demonstrating reproducible efficacy, manageable toxicity, and scalable manufacturing in comparative clinical trials.

## Conclusions and Outlook Toward a New Era in Solid Tumor Immunotherapy

Harnessing NK cells for solid tumors is steering the field toward a new phase of cancer immunotherapy. CAR-NK cells couple intrinsic safety with innate, multi-pronged cytotoxicity and thus offer a complementary or alternative platform to CAR-T. Preclinical and preliminary clinical studies demonstrate that engineered CAR-NK cells can circumvent selected immune evasion programs and achieve meaningful antitumor activity in solid tumor settings. Key innovations are broadening this potential. Armored constructs that supply autocrine survival cues, multi-specific and switchable designs that expand antigen coverage, and chemokine receptor engineering that improves homing are converging with rational combinations to dismantle microenvironmental barriers and enhance potency. Important hurdles remain, including antigen heterogeneity, physical and metabolic constraints within the tumor, and the need for durable persistence without added toxicity. Iterative optimization of targets, receptor architecture, delivery routes, and dosing schedules will be essential. Overall, CAR-NK therapy is advancing toward a safer and more effective modality that can be deployed across diverse solid tumors. Continued progress in engineering and combination strategies is likely to convert current proof of concept into durable clinical benefit and to improve outcomes for patients with refractory solid malignancies.

## References

[1] Berdeja JG, Madduri D, Usmani SZ, Jakubowiak A, Agha M, Cohen AD, Stewart AK, Hari P, Htut M, Lesokhin A, et al. Ciltacabtagene autoleucel, a B-cell maturation antigen-directed chimeric antigen receptor T-cell therapy in patients with relapsed or refractory multiple myeloma (CARTITUDE-1): A phase 1b/2 open-label study. Lancet. 2021;398(10297):314–324. 34175021 10.1016/S0140-6736(21)00933-8

[2] Maude SL, Frey N, Shaw PA, Aplenc R, Barrett DM, Bunin NJ, Chew A, Gonzalez VE, Zheng Z, Lacey SF, et al. Chimeric antigen receptor T cells for sustained remissions in leukemia. N Engl J Med. 2014;371(16):1507–1517. 25317870 10.1056/NEJMoa1407222 PMC4267531

[3] Neelapu SS, Locke FL, Bartlett NL, Lekakis LJ, Miklos DB, Jacobson CA, Braunschweig I, Oluwole OO, Siddiqi T, Lin Y, et al. Axicabtagene ciloleucel CAR T-cell therapy in refractory large B-cell lymphoma. N Engl J Med. 2017;377(26):2531–2544. 29226797 10.1056/NEJMoa1707447 PMC5882485

[4] Kochenderfer JN, Somerville RPT, Lu T, Yang JC, Sherry RM, Feldman SA, McIntyre L, Bot A, Rossi J, Lam N, et al. Long-duration complete remissions of diffuse large B cell lymphoma after anti-CD19 chimeric antigen receptor T cell therapy. Mol Ther. 2017;25(10):2245–2253. 28803861 10.1016/j.ymthe.2017.07.004 PMC5628864

[5] Myers JA, Miller JS. Exploring the NK cell platform for cancer immunotherapy. Nat Rev Clin Oncol. 2021;18(2):85–100. 32934330 10.1038/s41571-020-0426-7 PMC8316981

[6] Laskowski TJ, Biederstädt A, Rezvani K. Natural killer cells in antitumour adoptive cell immunotherapy. Nat Rev Cancer. 2022;22(10):557–575. 35879429 10.1038/s41568-022-00491-0 PMC9309992

[7] Liu E, Marin D, Banerjee P, Macapinlac HA, Thompson P, Basar R, Kerbauy LN, Overman B, Thall P, Kaplan M, et al. Use of CAR-transduced natural killer cells in CD19-positive lymphoid tumors. N Engl J Med. 2020;382(6):545–553. 32023374 10.1056/NEJMoa1910607 PMC7101242

[8] Becker PSA, Suck G, Nowakowska P, Ullrich E, Seifried E, Bader P, Tonn T, Seidl C. Selection and expansion of natural killer cells for NK cell-based immunotherapy. Cancer Immunol Immunother. 2016;65(4):477–484. 26810567 10.1007/s00262-016-1792-y PMC4826432

[9] Peng L, Sferruzza G, Yang L, Zhou L, Chen S. CAR-T and CAR-NK as cellular cancer immunotherapy for solid tumors. Cell Mol Immunol. 2024;21(10):1089–1108. 39134804 10.1038/s41423-024-01207-0 PMC11442786

[10] Binnewies M, Roberts EW, Kersten K, Chan V, Fearon DF, Merad M, Coussens LM, Gabrilovich DI, Ostrand-Rosenberg S, Hedrick CC, et al. Understanding the tumor immune microenvironment (TIME) for effective therapy. Nat Med. 2018;24(5):541–550. 29686425 10.1038/s41591-018-0014-x PMC5998822

[11] Vogelstein B, Papadopoulos N, Velculescu VE, Zhou S, Diaz LA, Kinzler KW. Cancer genome landscapes. Science. 2013;339(6127):1546–1558. 23539594 10.1126/science.1235122 PMC3749880

[12] Zhou Y, Cheng L, Liu L, Li X. NK cells are never alone: Crosstalk and communication in tumour microenvironments. Mol Cancer. 2023;22(1):34. 36797782 10.1186/s12943-023-01737-7 PMC9933398

[13] Azeez SS, Yashooa RK, Smail SW, Salihi A, Ali AS, Mamand S, Janson C., Advancing CAR-based cell therapies for solid tumours: Challenges, therapeutic strategies, and perspectives. Mol Cancer. 2025;24(1):191. 40624498 10.1186/s12943-025-02386-8 PMC12232864

[14] Ghaedrahmati F, Esmaeil N, Abbaspour M. Targeting immune checkpoints: How to use natural killer cells for fighting against solid tumors. Cancer Commun. 2023;43(2):177–213. 10.1002/cac2.12394 PMC9926962 36585761

[15] Chen S, Zhu H, Jounaidi Y. Comprehensive snapshots of natural killer cells functions, signaling, molecular mechanisms and clinical utilization. Signal Transduct Target Ther. 2024;9(1):302. 39511139 10.1038/s41392-024-02005-w PMC11544004

[16] Coënon L, Geindreau M, Ghiringhelli F, Villalba M, Bruchard M. Natural killer cells at the frontline in the fight against cancer. Cell Death Dis. 2024;15(8):614. 39179536 10.1038/s41419-024-06976-0 PMC11343846

[17] Sivori S, Vacca P, Del Zotto G, Munari E, Mingari MC, Moretta L. Human NK cells: Surface receptors, inhibitory checkpoints, and translational applications. Cell Mol Immunol. 2019;16(5):430–441. 30778167 10.1038/s41423-019-0206-4 PMC6474200

[18] Dean I, Lee CYC, Tuong ZK, Li Z, Tibbitt CA, Willis C, Gaspal F, Kennedy BC, Matei-Rascu V, Fiancette R, et al. Rapid functional impairment of natural killer cells following tumor entry limits anti-tumor immunity. Nat Commun. 2024;15(1):683. 38267402 10.1038/s41467-024-44789-z PMC10808449

[19] Egli L, Kaulfuss M, Mietz J, Picozzi A, Verhoeyen E, Münz C, Chijioke O. CAR T cells outperform CAR NK cells in CAR-mediated effector functions in head-to-head comparison. Exp Hematol Oncol. 2024;13(1):51. 38745250 10.1186/s40164-024-00522-6 PMC11092129

[20] Guedan S, Calderon H, Posey AD, Maus MV. Engineering and design of chimeric antigen receptors. Mol Ther Methods Clin Dev. 2018;12:145–156. 30666307 10.1016/j.omtm.2018.12.009 PMC6330382

[21] Lanier LL. DAP10- and DAP12-associated receptors in innate immunity. Immunol Rev. 2009;227(1):150–160. 19120482 10.1111/j.1600-065X.2008.00720.x PMC2794881

[22] Shah K, Al-Haidari A, Sun J, Kazi JU. T cell receptor (TCR) signaling in health and disease. Signal Transduct Target Ther. 2021;6(1):412. 34897277 10.1038/s41392-021-00823-w PMC8666445

[23] Yi E, Lee E, Park HJ, Lee HH, Yun SH, Kim HS. A chimeric antigen receptor tailored to integrate complementary activation signals potentiates the antitumor activity of NK cells. J Exp Clin Cancer Res. 2025;44(1):86. 40045373 10.1186/s13046-025-03351-5 PMC11884141

[24] Acharya S, Basar R, Daher M, Rafei H, Li P, Uprety N, Ensley E, Shanley M, Kumar B, Banerjee PP, et al. CD28 costimulation augments CAR signaling in NK cells via the LCK/CD3ζ/ZAP70 signaling axis. Cancer Discov. 2024;14(10):1879–1900. 38900051 10.1158/2159-8290.CD-24-0096 PMC11452288

[25] Xu Y, Liu Q, Zhong M, Wang Z, Chen Z, Zhang Y, Xing H, Tian Z, Tang K, Liao X, et al. 2B4 costimulatory domain enhancing cytotoxic ability of anti-CD5 chimeric antigen receptor engineered natural killer cells against T cell malignancies. J Hematol Oncol. 2019;12(1):49. 31097020 10.1186/s13045-019-0732-7 PMC6524286

[26] Rodriguez-Garcia A, Palazon A, Noguera-Ortega E, Powell DJ, Guedan S. CAR-T cells hit the tumor microenvironment: Strategies to overcome tumor escape. Front Immunol. 2020;11:1109. 32625204 10.3389/fimmu.2020.01109 PMC7311654

[27] Ghiringhelli F, Ménard C, Terme M, Flament C, Taieb J, Chaput N, Puig PE, Novault S, Escudier B, Vivier E, et al. CD4^+^CD25^+^ regulatory T cells inhibit natural killer cell functions in a transforming growth factor-beta-dependent manner. J Exp Med. 2005;202(8):1075–1085. 16230475 10.1084/jem.20051511 PMC2213209

[28] Smyth MJ, Teng MWL, Swann J, Kyparissoudis K, Godfrey DI, Hayakawa Y. CD4^+^CD25^+^ T regulatory cells suppress NK cell-mediated immunotherapy of cancer. J Immunol. 2006;176(3):1582–1587. 16424187 10.4049/jimmunol.176.3.1582

[29] Liu W, Wei X, Li L, Wu X, Yan J, Yang H, Song F. CCR4 mediated chemotaxis of regulatory T cells suppress the activation of T cells and NK cells via TGF-β pathway in human non-small cell lung cancer. Biochem Biophys Res Commun. 2017;488(1):196–203. 28487109 10.1016/j.bbrc.2017.05.034

[30] Shimizu J, Yamazaki S, Sakaguchi S. Induction of tumor immunity by removing CD25^+^CD4^+^ T cells: A common basis between tumor immunity and autoimmunity. J Immunol. 1999;163(10):5211–5218. 10553041

[31] Viel S, Marçais A, Guimaraes FS-F, Loftus R, Rabilloud J, Grau M, Degouve S, Djebali S, Salanville A, Charrier E, et al. TGF-β inhibits the activation and functions of NK cells by repressing the mTOR pathway. Sci Signal. 2016;9(415):ra19. 26884601 10.1126/scisignal.aad1884

[32] Du X, Moore J, Blank BR, Eksterowicz J, Sutimantanapi D, Yuen N, Metzger T, Chan B, Huang T, Chen X, et al. Orally bioavailable small-molecule CD73 inhibitor (OP-5244) reverses immunosuppression through blockade of adenosine production. J Med Chem. 2020;63(18):10433–10459. 32865411 10.1021/acs.jmedchem.0c01086

[33] Xia C, Yin S, To KKW, Fu L. CD39/CD73/A2AR pathway and cancer immunotherapy. Mol Cancer. 2023;22(1):44. 36859386 10.1186/s12943-023-01733-x PMC9979453

[34] Maj T, Wang W, Crespo J, Zhang H, Wang W, Wei S, Zhao L, Vatan L, Shao I, Szeliga W, et al. Oxidative stress controls regulatory T cell apoptosis and suppressor activity and PD-L1-blockade resistance in tumor. Nat Immunol. 2017;18(12):1332–1341. 29083399 10.1038/ni.3868 PMC5770150

[35] Pandiyan P, Zheng L, Ishihara S, Reed J, Lenardo MJ. CD4^+^CD25^+^Foxp3^+^ regulatory T cells induce cytokine deprivation-mediated apoptosis of effector CD4^+^ T cells. Nat Immunol. 2007;8(12):1353–1362. 17982458 10.1038/ni1536

[36] Jie H-B, Schuler PJ, Lee SC, Srivastava RM, Argiris A, Ferrone S, et al. CTLA-4^+^ regulatory T cells increased in cetuximab-treated head and neck cancer patients suppress NK cell cytotoxicity and correlate with poor prognosis. Cancer Res. 2015;75(11):2200–2210. 25832655 10.1158/0008-5472.CAN-14-2788 PMC4452385

[37] Veglia F, Sanseviero E, Gabrilovich DI. Myeloid-derived suppressor cells in the era of increasing myeloid cell diversity. Nat Rev Immunol. 2021;21(8):485–498. 33526920 10.1038/s41577-020-00490-y PMC7849958

[38] Li H, Han Y, Guo Q, Zhang M, Cao X. Cancer-expanded myeloid-derived suppressor cells induce anergy of NK cells through membrane-bound TGF-β1. J Immunol. 2009;182(1):240–249. 19109155 10.4049/jimmunol.182.1.240

[39] Hoechst B, Voigtlaender T, Ormandy L, Gamrekelashvili J, Zhao F, Wedemeyer H, Lehner F, Manns MP, Greten TF, Korangy F. Myeloid derived suppressor cells inhibit natural killer cells in patients with hepatocellular carcinoma via the NKp30 receptor. Hepatology. 2009;50(3):799–807. 19551844 10.1002/hep.23054 PMC6357774

[40] Stiff A, Trikha P, Mundy-Bosse B, McMichael E, Mace TA, Benner B, Kendra K, Campbell A, Gautam S, Abood D, et al. Nitric oxide production by myeloid-derived suppressor cells plays a role in impairing Fc receptor-mediated natural killer cell function. Clin Cancer Res. 2018;24(8):1891–1904. 29363526 10.1158/1078-0432.CCR-17-0691 PMC7184799

[41] Zhang J, Han X, Hu X, Jin F, Gao Z, Yin L, et al. IDO1 impairs NK cell cytotoxicity by decreasing NKG2D/NKG2DLs via promoting miR-18a. Mol Immunol. 2018;103:144–155. 30268986 10.1016/j.molimm.2018.09.011

[42] He S, Zheng L, Qi C. Myeloid-derived suppressor cells (MDSCs) in the tumor microenvironment and their targeting in cancer therapy. Mol Cancer. 2025;24(1):5. 39780248 10.1186/s12943-024-02208-3 PMC11707952

[43] Greene S, Robbins Y, Mydlarz WK, Huynh AP, Schmitt NC, Friedman J, Horn LA, Palena C, Schlom J, Maeda DY, et al. Inhibition of MDSC trafficking with SX-682, a CXCR1/2 inhibitor, enhances NK-cell immunotherapy in head and neck cancer models. Clin Cancer Res. 2020;26(6):1420–1431. 31848188 10.1158/1078-0432.CCR-19-2625 PMC7073293

[44] Koinis F, Vetsika EK, Aggouraki D, Skalidaki E, Koutoulaki A, Gkioulmpasani M, Georgoulias V, Kotsakis A. Effect of first-line treatment on myeloid-derived suppressor cells’ subpopulations in the peripheral blood of patients with non-small cell lung cancer. J Thorac Oncol. 2016;11(8):1263–1272. 27178984 10.1016/j.jtho.2016.04.026

[45] Alizadeh D, Trad M, Hanke NT, Larmonier CB, Janikashvili N, Bonnotte B, Katsanis E, Larmonier N. Doxorubicin eliminates myeloid-derived suppressor cells and enhances the efficacy of adoptive T-cell transfer in breast cancer. Cancer Res. 2014;74(1):104–118. 24197130 10.1158/0008-5472.CAN-13-1545 PMC3896092

[46] Suzuki E, Kapoor V, Jassar AS, Kaiser LR, Albelda SM. Gemcitabine selectively eliminates splenic Gr-1^+^/CD11b^+^ myeloid suppressor cells in tumor-bearing animals and enhances antitumor immune activity. Clin Cancer Res. 2005;11(18):6713–6721. 16166452 10.1158/1078-0432.CCR-05-0883

[47] Mao Y, Sarhan D, Steven A, Seliger B, Kiessling R, Lundqvist A. Inhibition of tumor-derived prostaglandin-e2 blocks the induction of myeloid-derived suppressor cells and recovers natural killer cell activity. Clin Cancer Res. 2014;20(15):4096–4106. 24907113 10.1158/1078-0432.CCR-14-0635

[48] Li K, Shi H, Zhang B, Ou X, Ma Q, Chen Y, Shu P, Li D, Wang Y. Myeloid-derived suppressor cells as immunosuppressive regulators and therapeutic targets in cancer. Signal Transduct Target Ther. 2021;6(1):362. 34620838 10.1038/s41392-021-00670-9 PMC8497485

[49] Noy R, Pollard JW. Tumor-associated macrophages: From mechanisms to therapy. Immunity. 2014;41(1):49–61. 25035953 10.1016/j.immuni.2014.06.010 PMC4137410

[50] Nuñez SY, Ziblat A, Secchiari F, Torres NI, Sierra JM, Raffo Iraolagoitia XL, Araya RE, Domaica CI, Fuertes MB, Zwirner NW. Human M2 macrophages limit NK cell effector functions through secretion of TGF-β and engagement of CD85j. J Immunol. 2018;200(3):1008–1015. 29282306 10.4049/jimmunol.1700737

[51] Wu Y, Kuang DM, Pan WD, Wan YL, Lao XM, Wang D, Li XF, Zheng L. Monocyte/macrophage-elicited natural killer cell dysfunction in hepatocellular carcinoma is mediated by CD48/2B4 interactions. Hepatology. 2013;57(3):1107–1116. 23225218 10.1002/hep.26192

[52] Eisinger S, Sarhan D, Boura VF, Ibarlucea-Benitez I, Tyystjärvi S, Oliynyk G, Arsenian-Henriksson M, Lane D, Wikström SL, Kiessling R, et al. Targeting a scavenger receptor on tumor-associated macrophages activates tumor cell killing by natural killer cells. Proc Natl Acad Sci USA. 2020;117(50):32005–32016. 33229588 10.1073/pnas.2015343117 PMC7750482

[53] Liu T, Han C, Wang S, Fang P, Ma Z, Xu L, Yin R. Cancer-associated fibroblasts: An emerging target of anti-cancer immunotherapy. J Hematol Oncol. 2019;12(1):86. 31462327 10.1186/s13045-019-0770-1 PMC6714445

[54] Li T, Yang Y, Hua X, Wang G, Liu W, Jia C, Tai Y, Zhang Q, Chen G. Hepatocellular carcinoma-associated fibroblasts trigger NK cell dysfunction via PGE2 and IDO. Cancer Lett. 2012;318(2):154–161. 22182446 10.1016/j.canlet.2011.12.020

[55] Li T, Yi S, Liu W, Jia C, Wang G, Hua X, Tai Y, Zhang Q, Chen G. Colorectal carcinoma-derived fibroblasts modulate natural killer cell phenotype and antitumor cytotoxicity. Med Oncol. 2013;30(3):663. 23873014 10.1007/s12032-013-0663-z

[56] Balsamo M, Scordamaglia F, Pietra G, Manzini C, Cantoni C, Boitano M, Queirolo P, Vermi W, Facchetti F, Moretta A, et al. Melanoma-associated fibroblasts modulate NK cell phenotype and antitumor cytotoxicity. Proc Natl Acad Sci USA. 2009;106(49):20847–20852. 19934056 10.1073/pnas.0906481106 PMC2791633

[57] Inoue T, Adachi K, Kawana K, Taguchi A, Nagamatsu T, Fujimoto A, Tomio K, Yamashita A, Eguchi S, Nishida H, et al. Cancer-associated fibroblast suppresses killing activity of natural killer cells through downregulation of poliovirus receptor (PVR/CD155), a ligand of activating NK receptor. Int J Oncol. 2016;49(4):1297–1304. 27499237 10.3892/ijo.2016.3631 PMC5021244

[58] Ireland L, Luckett T, Schmid MC, Mielgo A. Blockade of stromal Gas6 alters cancer cell plasticity, activates NK cells, and inhibits pancreatic cancer metastasis. Front Immunol. 2020;11:297. 32174917 10.3389/fimmu.2020.00297 PMC7056881

[59] Costa D, Venè R, Benelli R, Romairone E, Scabini S, Catellani S, Rebesco B, Mastracci L, Grillo F, Minghelli S, et al. Targeting the epidermal growth factor receptor can counteract the inhibition of natural killer cell function exerted by colorectal tumor-associated fibroblasts. Front Immunol. 2018;9:1150. 29910806 10.3389/fimmu.2018.01150 PMC5992415

[60] Parodi M, Raggi F, Cangelosi D, Manzini C, Balsamo M, Blengio F, Eva A, Varesio L, Pietra G, Moretta L, et al. Hypoxia modifies the transcriptome of human NK cells, modulates their immunoregulatory profile, and influences NK cell subset migration. Front Immunol. 2018;9:2358. 30459756 10.3389/fimmu.2018.02358 PMC6232835

[61] Zheng X, Qian Y, Fu B, Jiao D, Jiang Y, Chen P, Shen Y, Zhang H, Sun R, Tian Z, et al. Mitochondrial fragmentation limits NK cell-based tumor immunosurveillance. Nat Immunol. 2019;20(12):1656–1667. 31636463 10.1038/s41590-019-0511-1

[62] Balsamo M, Manzini C, Pietra G, Raggi F, Blengio F, Mingari MC, Varesio L, Moretta L, Bosco MC, Vitale M. Hypoxia downregulates the expression of activating receptors involved in NK-cell-mediated target cell killing without affecting ADCC. Eur J Immunol. 2013;43(10):2756–2764. 23913266 10.1002/eji.201343448

[63] Baginska J, Viry E, Berchem G, Poli A, Noman MZ, van Moer K, Medves S, Zimmer J, Oudin A, Niclou SP, et al. Granzyme B degradation by autophagy decreases tumor cell susceptibility to natural killer-mediated lysis under hypoxia. Proc Natl Acad Sci USA. 2013;110(43):17450–17455. 24101526 10.1073/pnas.1304790110 PMC3808626

[64] Raskovalova T, Huang X, Sitkovsky M, Zacharia LC, Jackson EK, Gorelik E. Gs protein-coupled adenosine receptor signaling and lytic function of activated NK cells. J Immunol. 2005;175(7):4383–4391. 16177079 10.4049/jimmunol.175.7.4383

[65] Raskovalova T, Lokshin A, Huang X, Jackson EK, Gorelik E. Adenosine-mediated inhibition of cytotoxic activity and cytokine production by IL-2/NKp46-activated NK cells: Involvement of protein kinase A isozyme I (PKAI). Immunol Res. 2006;36(1):91–99. 17337770 10.1385/IR:36:1:91

[66] Santoni G, Amantini C, Santoni M, Maggi F, Morelli MB, Santoni A. Mechanosensation and mechanotransduction in natural killer cells. Front Immunol. 2021;12: Article 688918. 34335592 10.3389/fimmu.2021.688918 PMC8320435

[67] Henke E, Nandigama R, Ergün S. Extracellular matrix in the tumor microenvironment and its impact on cancer therapy. Front Mol Biosci. 2019;6:160. 32118030 10.3389/fmolb.2019.00160 PMC7025524

[68] Prakash J, Shaked Y. The interplay between extracellular matrix remodeling and cancer therapeutics. Cancer Discov. 2024;14(8):1375–1388. 39091205 10.1158/2159-8290.CD-24-0002 PMC11294818

[69] Mao X, Xu J, Wang W, Liang C, Hua J, Liu J, Zhang B, Meng Q, Yu X, Shi S. Crosstalk between cancer-associated fibroblasts and immune cells in the tumor microenvironment: New findings and future perspectives. Mol Cancer. 2021;20(1):131. 34635121 10.1186/s12943-021-01428-1 PMC8504100

[70] Sarntinoranont M, Rooney F, Ferrari M. Interstitial stress and fluid pressure within a growing tumor. Ann Biomed Eng. 2003;31(3):327–335. 12680730 10.1114/1.1554923

[71] Barkovskaya A, Buffone A, Žídek M, Weaver VM. Proteoglycans as mediators of cancer tissue mechanics. Front Cell Dev Biol. 2020;8: Article 569377. 33330449 10.3389/fcell.2020.569377 PMC7734320

[72] Walker C, Mojares E, Del Río Hernández A. Role of extracellular matrix in development and cancer progression. Int J Mol Sci. 2018;19(10): Article 3028. 30287763 10.3390/ijms19103028 PMC6213383

[73] Lugano R, Ramachandran M, Dimberg A. Tumor angiogenesis: Causes, consequences, challenges and opportunities. Cell Mol Life Sci. 2020;77(9):1745–1770. 31690961 10.1007/s00018-019-03351-7 PMC7190605

[74] Zimna A, Kurpisz M. Hypoxia-inducible factor-1 in physiological and pathophysiological angiogenesis: Applications and therapies. Biomed Res Int. 2015;2015(1): Article 549412. 26146622 10.1155/2015/549412 PMC4471260

[75] Harjunpää H, Llort Asens M, Guenther C, Fagerholm SC. Cell adhesion molecules and their roles and regulation in the immune and tumor microenvironment. Front Immunol. 2019;10:1078. 31231358 10.3389/fimmu.2019.01078 PMC6558418

[76] Ran GH, Lin YQ, Tian L, Zhang T, Yan DM, Yu JH, Deng YC. Natural killer cell homing and trafficking in tissues and tumors: From biology to application. Signal Transduct Target Ther. 2022;7(1):205. 35768424 10.1038/s41392-022-01058-z PMC9243142

[77] Rezaeifard S, Talei A, Shariat M, Erfani N. Tumor infiltrating NK cell (TINK) subsets and functional molecules in patients with breast cancer. Mol Immunol. 2021;136:161–167. 34171565 10.1016/j.molimm.2021.03.003

[78] Tomaipitinca L, Russo E, Bernardini G. NK cell surveillance of hematological malignancies. Therapeutic implications and regulation by chemokine receptors. Mol Asp Med. 2021;80: Article 100968. 10.1016/j.mam.2021.100968 34045078

[79] Mroz EA, Rocco JW. The challenges of tumor genetic diversity. Cancer. 2017;123(6):917–927. 27861749 10.1002/cncr.30430 PMC5370554

[80] Cózar B, Greppi M, Carpentier S, Narni-Mancinelli E, Chiossone L, Vivier E. Tumor-infiltrating natural killer cells. Cancer Discov. 2020;11(1):34–44. 33277307 10.1158/2159-8290.CD-20-0655 PMC7611243

[81] Zhang X, Rao A, Sette P, Deibert C, Pomerantz A, Kim WJ, Kohanbash G, Chang Y, Park Y, Engh J, et al. IDH mutant gliomas escape natural killer cell immune surveillance by downregulation of NKG2D ligand expression. Neuro-Oncology. 2016;18(10):1402–1412. 27116977 10.1093/neuonc/now061 PMC5035522

[82] Veeraraghavan VP, Doni BR, Dasari AK, Patil C, Rao KA, Patil SR. Deciphering genomic complexity: Understanding intratumor heterogeneity, clonal evolution, and therapeutic vulnerabilities in oral squamous cell carcinoma. Oral Oncol Rep. 2024;10: Article 100469.

[83] Cao J, Yan Q. Cancer epigenetics, tumor immunity, and immunotherapy. Trends Cancer. 2020;6(7):580–592. 32610068 10.1016/j.trecan.2020.02.003 PMC7330177

[84] Vyas M, Müller R, Pogge von Strandmann E. Antigen loss variants: Catching hold of escaping foes. Front Immunol. 2017;8:175. 28286501 10.3389/fimmu.2017.00175 PMC5323381

[85] Miller JS, Soignier Y, Panoskaltsis-Mortari A, McNearney SA, Yun GH, Fautsch SK, McKenna D, Le C, Defor TE, Burns LJ, et al. Successful adoptive transfer and in vivo expansion of human haploidentical NK cells in patients with cancer. Blood. 2005;105(8):3051–3057. 15632206 10.1182/blood-2004-07-2974

[86] Bachanova V, Cooley S, Defor TE, Verneris MR, Zhang B, McKenna DH, Curtsinger J, Panoskaltsis-Mortari A, Lewis D, Hippen K, et al. Clearance of acute myeloid leukemia by haploidentical natural killer cells is improved using IL-2 diphtheria toxin fusion protein. Blood. 2014;123(25):3855–3863. 24719405 10.1182/blood-2013-10-532531 PMC4064329

[87] Barshidi A, Ardeshiri K, Ebrahimi F, Alian F, Shekarchi AA, Hojjat-Farsangi M, Jadidi-Niaragh F. The role of exhausted natural killer cells in the immunopathogenesis and treatment of leukemia. Cell Commun Signal. 2024;22(1):59. 38254135 10.1186/s12964-023-01428-2 PMC10802000

[88] Bi J, Tian Z. NK cell exhaustion. Front Immunol. 2017;8:760. 28702032 10.3389/fimmu.2017.00760 PMC5487399

[89] Beldi-Ferchiou A, Lambert M, Dogniaux S, Vély F, Vivier E, Olive D, Dupuy S, Levasseur F, Zucman D, Lebbe C, et al. PD-1 mediates functional exhaustion of activated NK cells in patients with Kaposi sarcoma. Oncotarget. 2016;7(45):72961–72977. 27662664 10.18632/oncotarget.12150 PMC5341956

[90] Wiesmayr S, Webber SA, Macedo C, Popescu I, Smith L, Luce J, Metes D. Decreased NKp46 and NKG2D and elevated PD-1 are associated with altered NK-cell function in pediatric transplant patients with PTLD. Eur J Immunol. 2012;42(2):541–550. 22105417 10.1002/eji.201141832 PMC3607363

[91] Terrén I, Orrantia A, Vitallé J, Zenarruzabeitia O, Borrego F. NK cell metabolism and tumor microenvironment. Front Immunol. 2019;10:2278. 31616440 10.3389/fimmu.2019.02278 PMC6769035

[92] Wei J, Han X, Bo J, Han W. Target selection for CAR-T therapy. J Hematol Oncol. 2019;12(1):62. 31221182 10.1186/s13045-019-0758-x PMC6587237

[93] Rafiq S, Hackett CS, Brentjens RJ. Engineering strategies to overcome the current roadblocks in CAR T cell therapy. Nat Rev Clin Oncol. 2020;17(3):147–167. 31848460 10.1038/s41571-019-0297-y PMC7223338

[94] Morgan RA, Yang JC, Kitano M, Dudley ME, Laurencot CM, Rosenberg SA. Case report of a serious adverse event following the administration of T cells transduced with a chimeric antigen receptor recognizing ERBB2. Mol Ther. 2010;18(4):843–851. 20179677 10.1038/mt.2010.24 PMC2862534

[95] Haas AR, Golden RJ, Litzky LA, Engels B, Zhao L, Xu F, Taraszka JA, Ramones M, Granda B, Chang WJ, et al. Two cases of severe pulmonary toxicity from highly active mesothelin-directed CAR T cells. Mol Ther. 2023;31(8):2309–2325. 37312454 10.1016/j.ymthe.2023.06.006 PMC10422001

[96] Wang K, Wang L, Wang Y, Xiao L, Wei J, Hu Y, Wang D, Huang H. Reprogramming natural killer cells for cancer therapy. Mol Ther. 2024;32(9):2835–2855. 38273655 10.1016/j.ymthe.2024.01.027 PMC11403237

[97] Tang X, Yang L, Li Z, Nalin AP, Dai H, Xu T, Yin J, You F, Zhu M, Shen W, et al. First-in-man clinical trial of CAR NK-92 cells: Safety test of CD33-CAR NK-92 cells in patients with relapsed and refractory acute myeloid leukemia. Am J Cancer Res. 2018;8(6):1083–1089. 30034945 PMC6048396

[98] Joshi S, Sharabi A. Targeting myeloid-derived suppressor cells to enhance natural killer cell-based immunotherapy. Pharmacol Ther. 2022;235: Article 108114. 35122833 10.1016/j.pharmthera.2022.108114 PMC9189042

[99] Hosseinalizadeh H, Wang L-S, Mirzaei H, Amoozgar Z, Tian L, Yu J. Emerging combined CAR-NK cell therapies in cancer treatment: Finding a dancing partner. Mol Ther. 2025;33(6):2406–2425. 39754357 10.1016/j.ymthe.2024.12.057 PMC12172187

[100] MacDonald KP, Palmer JS, Cronau S, Seppanen E, Olver S, Raffelt NC, Kuns R, Pettit AR, Clouston A, Wainwright B, et al. An antibody against the colony-stimulating factor 1 receptor depletes the resident subset of monocytes and tissue- and tumor-associated macrophages but does not inhibit inflammation. Blood. 2010;116(19):3955–3963. 20682855 10.1182/blood-2010-02-266296

[101] Lian G, Mak TS-K, Yu X, Lan H-Y. Challenges and recent advances in NK cell-targeted immunotherapies in solid tumors. Int J Mol Sci. 2021;23(1): Article 164. 35008589 10.3390/ijms23010164 PMC8745474

[102] Kilgour MK, Bastin DJ, Lee S-H, Ardolino M, McComb S, Visram A. Advancements in CAR-NK therapy: Lessons to be learned from CAR-T therapy. Front Immunol. 2023;14:1166038. 37205115 10.3389/fimmu.2023.1166038 PMC10187144

[103] Trotta R, Col JD, Yu J, Ciarlariello D, Thomas B, Zhang X, Allard J, Wei M, Mao H, Byrd JC, et al. TGF-beta utilizes SMAD3 to inhibit CD16-mediated IFN-gamma production and antibody-dependent cellular cytotoxicity in human NK cells. J Immunol. 2008;181(6):3784–3792. 18768831 10.4049/jimmunol.181.6.3784 PMC2924753

[104] Friese MA, Wischhusen J, Wick W, Weiler M, Eisele G, Steinle A, Weller M. RNA interference targeting transforming growth factor-beta enhances NKG2D-mediated antiglioma immune response, inhibits glioma cell migration and invasiveness, and abrogates tumorigenicity in vivo. Cancer Res. 2004;64(20):7596–7603. 15492287 10.1158/0008-5472.CAN-04-1627

[105] Crane CA, Han SJ, Barry JJ, Ahn BJ, Lanier LL, Parsa AT. TGF-beta downregulates the activating receptor NKG2D on NK cells and CD8^+^ T cells in glioma patients. Neuro-Oncology. 2010;12(1):7–13. 20150362 10.1093/neuonc/nop009 PMC2940557

[106] Thangaraj JL, Coffey M, Lopez E, Kaufman DS. Disruption of TGF-β signaling pathway is required to mediate effective killing of hepatocellular carcinoma by human iPSC-derived NK cells. Cell Stem Cell. 2024;31(9):1327–1343.e5. 38986609 10.1016/j.stem.2024.06.009 PMC11380586

[107] Oh M-S, Seo M, Yoon S, Jang M. 292 Unleashing CAR-NK cells against solid tumors by disrupting TGF-β signaling in the tumor microenvironment. J ImmunoTher Cancer. 2023;11(1).

[108] Shin SH, Lee YE, Yoon HN, Yuk CM, An JY, Seo M, Yoon S, Oh MS, Shin SC, Kim JH, et al. An innovative strategy harnessing self-activating CAR-NK cells to mitigate TGF-β1-driven immune suppression. Biomaterials. 2025;314: Article 122888. 39423512 10.1016/j.biomaterials.2024.122888

[109] Klopotowska M, Bajor M, Graczyk-Jarzynka A, Kraft A, Pilch Z, Zhylko A, Firczuk M, Baranowska I, Lazniewski M, Plewczynski D, et al. PRDX-1 supports the survival and antitumor activity of primary and CAR-modified NK cells under oxidative stress. Cancer Immunol Res. 2022;10(2):228–244. 34853030 10.1158/2326-6066.CIR-20-1023 PMC9414282

[110] Ni J, Wang X, Stojanovic A, Zhang Q, Wincher M, Bühler L, Arnold A, Correia MP, Winkler M, Koch PS, et al. Single-cell RNA sequencing of tumor-infiltrating NK cells reveals that inhibition of transcription factor HIF-1α unleashes NK cell activity. Immunity. 2020;52(6):1075–1087. 32445619 10.1016/j.immuni.2020.05.001

[111] Juillerat A, Marechal A, Filhol JM, Valogne Y, Valton J, Duclert A, Duchateau P, Poirot L. An oxygen sensitive self-decision making engineered CAR T-cell. Sci Rep. 2017;7(1):39833. 28106050 10.1038/srep39833 PMC5247770

[112] Young A, Ngiow SF, Gao Y, Patch AM, Barkauskas DS, Messaoudene M, Lin G, Coudert JD, Stannard KA, Zitvogel L, et al. A2AR adenosine signaling suppresses natural killer cell maturation in the tumor microenvironment. Cancer Res. 2018;78(4):1003–1016. 29229601 10.1158/0008-5472.CAN-17-2826

[113] Nachef M, Ali AK, Almutairi SM, Lee S-H. Targeting SLC1A5 and SLC3A2/SLC7A5 as a potential strategy to strengthen anti-tumor immunity in the tumor microenvironment. Front Immunol. 2021;12: Article 624324. 33953707 10.3389/fimmu.2021.624324 PMC8089370

[114] Lin X, Liu Z, Dong X, Wang K, Sun Y, Zhang H, Wang F, Chen Y, Ling J, Guo Y, et al. Radiotherapy enhances the anti-tumor effect of CAR-NK cells for hepatocellular carcinoma. J Transl Med. 2024;22(1):929. 39396988 10.1186/s12967-024-05724-4 PMC11472550

[115] Ng YY, Tay JCK, Wang S. CXCR1 expression to improve anti-cancer efficacy of intravenously injected CAR-NK cells in mice with peritoneal xenografts. Mol Ther Oncolytics. 2019;16:75–85. 31970285 10.1016/j.omto.2019.12.006 PMC6965500

[116] Müller N, Michen S, Tietze S, Töpfer K, Schulte A, Lamszus K, Schmitz M, Schackert G, Pastan I, Temme A, et al. Engineering NK cells modified with an EGFRvIII-specific chimeric antigen receptor to overexpress CXCR4 improves immunotherapy of CXCL12/SDF-1α-secreting glioblastoma. J Immunother. 2015;38(5):197–210. 25962108 10.1097/CJI.0000000000000082 PMC4428685

[117] Li F, Sheng Y, Hou W, Sampath P, Byrd D, Thorne S, et al. CCL5-armed oncolytic virus augments CCR5-engineered NK cell infiltration and antitumor efficiency. J ImmunoTher Cancer. 2020;8(1): Article e000131. 32098828 10.1136/jitc-2019-000131 PMC7057442

[118] Lee J, Kang TH, Yoo W, Choi H, Jo S, Kong K, Lee SR, Kim SU, Kim JS, Cho D, et al. An antibody designed to improve adoptive NK-cell therapy inhibits pancreatic cancer progression in a murine model. Cancer Immunol Res. 2019;7(2):219–229. 30514792 10.1158/2326-6066.CIR-18-0317

[119] Caruana I, Savoldo B, Hoyos V, Weber G, Liu H, Kim ES, Ittmann MM, Marchetti D, Dotti G. Heparanase promotes tumor infiltration and antitumor activity of CAR-redirected T lymphocytes. Nat Med. 2015;21(5):524–529. 25849134 10.1038/nm.3833 PMC4425589

[120] Liborio-Ramos S, Quiros-Fernandez I, Ilan N, Soboh S, Farhoud M, Süleymanoglu R, Bennek M, Calleja-Vara S, Müller M, Vlodavsky I, et al. An integral membrane constitutively active heparanase enhances the tumor infiltration capability of NK cells. Onco Targets Ther. 2025;14(1):2437917. 10.1080/2162402X.2024.2437917 PMC11633225 39651893

[121] Shen R, Peng L, Zhou W, Wang D, Jiang Q, Ji J, Hu F, Yuan H. Anti-angiogenic nano-delivery system promotes tumor vascular normalizing and micro-environment reprogramming in solid tumor. J Control Release. 2022;349:550–564. 35841997 10.1016/j.jconrel.2022.07.015

[122] Zhao Y, Yu X, Li J. Manipulation of immune–vascular crosstalk: New strategies towards cancer treatment. Acta Pharm Sin B. 2020;10(11):2018–2036. 33304777 10.1016/j.apsb.2020.09.014 PMC7714955

[123] Shrimali RK, Yu Z, Theoret MR, Chinnasamy D, Restifo NP, Rosenberg SA. Antiangiogenic agents can increase lymphocyte infiltration into tumor and enhance the effectiveness of adoptive immunotherapy of cancer. Cancer Res. 2010;70(15):6171–6180. 20631075 10.1158/0008-5472.CAN-10-0153 PMC2912959

[124] Dong X, Ren J, Amoozgar Z, Lee S, Datta M, Roberge S, Duquette M, Fukumura D, Jain RK. Anti-VEGF therapy improves EGFR-vIII-CAR-T cell delivery and efficacy in syngeneic glioblastoma models in mice. J immunoTher Cancer. 2023;11(3): Article e005583. 36898734 10.1136/jitc-2022-005583 PMC10008211

[125] Zhang C, Burger MC, Jennewein L, Genßler S, Schönfeld K, Zeiner P, Hattingen E, Harter PN, Mittelbronn M, Tonn T, et al. ErbB2/HER2-specific NK cells for targeted therapy of glioblastoma. J Natl Cancer Inst. 2016;108(5): Article djc375. 10.1093/jnci/djv375 26640245

[126] Xiao L, Cen D, Gan H, Sun Y, Huang N, Xiong H, Jin Q, Su L, Liu X, Wang K, et al. Adoptive transfer of NKG2D CAR mRNA-engineered natural killer cells in colorectal cancer patients. Mol Ther. 2019;27(6):1114–1125. 30962163 10.1016/j.ymthe.2019.03.011 PMC6554529

[127] Adotevi O, Godet Y, Galaine J, Lakkis Z, Idirene I, Certoux JM, Jary M, Loyon R, Laheurte C, Kim S, et al. In situ delivery of allogeneic natural killer cell (NK) combined with Cetuximab in liver metastases of gastrointestinal carcinoma: A phase I clinical trial. Onco Targets Ther. 2018;7(5): Article e1424673. 10.1080/2162402X.2018.1424673 PMC5927529 29721386

[128] Wu M, He J, Geng J, Wei Z, Cheng R, Li H, Xing L. Robo1 CAR-NK92 and radiotherapy exert synergistic efficacy in solid tumors. J Transl Med. 2025;23(1):720. 40597275 10.1186/s12967-025-06753-3 PMC12218003

[129] Noordam L, Kaijen ME, Bezemer K, Cornelissen R, Maat LA, Hoogsteden HC, Aerts JG, Hendriks RW, Hegmans JP, Vroman H. Low-dose cyclophosphamide depletes circulating naïve and activated regulatory T cells in malignant pleural mesothelioma patients synergistically treated with dendritic cell-based immunotherapy. Onco Targets Ther. 2018;7(12): Article e1474318. 10.1080/2162402X.2018.1474318 PMC6279421 30524884

[130] Law AMK, Valdes-Mora F, Gallego-Ortega D. Myeloid-derived suppressor cells as a therapeutic target for cancer. Cells. 2020;9(3): Article 561. 32121014 10.3390/cells9030561 PMC7140518

[131] Sugahara KN, Teesalu T, Karmali PP, Kotamraju VR, Agemy L, Girard OM, Hanahan D, Mattrey RF, Ruoslahti E. Tissue-penetrating delivery of compounds and nanoparticles into tumors. Cancer Cell. 2009;16(6):510–520. 19962669 10.1016/j.ccr.2009.10.013 PMC2791543

[132] Dong Y, Huang Y, Zhang Z, Chen A, Li L, Tian M, Shen J, Shao J. iRGD-modified memory-like NK cells exhibit potent responses to hepatocellular carcinoma. J Transl Med. 2023;21(1):205. 36932395 10.1186/s12967-023-04024-7 PMC10022190

[133] Song G, Qi X, Zhao Y. iRGD tumor penetrating peptide-modified NK cells exhibit enhanced tumor immune infiltration ability and anti-tumor efficacy. Protein Pept Lett. 2025;32(3):183–193. 39950266 10.2174/0109298665348639250115113650 PMC12307954

[134] Bielamowicz K, Fousek K, Byrd TT, Samaha H, Mukherjee M, Aware N, Wu MF, Orange JS, Sumazin P, Man TK, et al. Trivalent CAR T cells overcome interpatient antigenic variability in glioblastoma. Neuro-Oncology. 2018;20(4):506–518. 29016929 10.1093/neuonc/nox182 PMC5909636

[135] Kim H, Han M, Kim M, Kim H, Im HJ, Kim N, Koh KN. CD19/CD22 bispecific chimeric antigen receptor-NK-92 cells are developed and evaluated. Oncol Lett. 2023;25(6):236. 37153038 10.3892/ol.2023.13822 PMC10161343

[136] Junca AG, Frankel N, Gainer M, Mullenix A, Palermo M, Lee D, Liu F, Gordley R, Lee CT, Roguev A, et al. 116 Development of logic gated CAR-NK cells for the treatment of solid tumors. J ImmunoTher Cancer. 2021;9(2).

[137] Roex G, Campillo-Davo D, Flumens D, Shaw PA, Krekelbergh L, De Reu H, Berneman ZN, Lion E, Anguille S. Two for one: Targeting BCMA and CD19 in B-cell malignancies with off-the-shelf dual-CAR NK-92 cells. J Transl Med. 2022;20(1):124. 35287669 10.1186/s12967-022-03326-6 PMC8919645

[138] Zhi L, Zhang Z, Gao Q, Shang C, He W, Wang Y, Guo C, Niu Z, Zhu W. CAR-NK cells with dual targeting of PD-L1 and MICA/B in lung cancer tumor models. BMC Cancer. 2025;25(1):337. 40000974 10.1186/s12885-025-13780-2 PMC11853679

[139] Wang Y, Zheng X, Wang Z, Xiao Z, Lin Y, Zhang F, Liu Y, Liu P, Weng Q, Zhang L, et al. Avoidance of fratricide and improved efficacy of CD33-MSLN CAR-iNK cells in treating acute myeloid leukemia. bioRxiv. 2025. 10.1101/2025.01.23.634500

[140] Mohammad A, Yurina A, Simonyan T, Chistyakov D, Salman R, Zornikova K, Minina E, Bogolyubova A. Modular (universal) CAR-T platforms in vivo: A comprehensive systematic review. Front Immunol. 2024;15:1409665. 39712013 10.3389/fimmu.2024.1409665 PMC11659234

[141] Mitwasi N, Feldmann A, Arndt C, Koristka S, Berndt N, Jureczek J, Loureiro LR, Bergmann R, Máthé D, Hegedüs N, et al. “UniCAR”-modified off-the-shelf NK-92 cells for targeting of GD2-expressing tumour cells. Sci Rep. 2020;10(1):2141. 32034289 10.1038/s41598-020-59082-4 PMC7005792

[142] Pfeifer Serrahima J, Zhang C, Oberoi P, Bodden M, Röder J, Arndt C, Feldmann A, Kiefer A, Prüfer M, Kühnel I, et al. Multivalent adaptor proteins specifically target NK cells carrying a universal chimeric antigen receptor to ErbB2 (HER2)-expressing cancers. Cancer Immunol Immunother. 2023;72(9):2905–2918. 36688995 10.1007/s00262-023-03374-x PMC10412657

[143] Grote S, Mittelstaet J, Baden C, Chan KC, Seitz C, Schlegel P, Kaiser A, Handgretinger R, Schleicher S. Adapter chimeric antigen receptor (AdCAR)-engineered NK-92 cells for the multiplex targeting of bone metastases. Cancer. 2021;13(5): Article 1825177. 10.3390/cancers13051124 PMC7961358 33807875

[144] Grote S, Mittelstaet J, Baden C, Chan KC, Seitz C, Schlegel P, Kaiser A, Handgretinger R, Schleicher S. Adapter chimeric antigen receptor (AdCAR)-engineered NK-92 cells: An off-the-shelf cellular therapeutic for universal tumor targeting. Onco Targets Ther. 2020;9(1): Article 1825177. 10.1080/2162402X.2020.1825177 PMC7781805 33457105

[145] Landgraf KE, Williams SR, Steiger D, Gebhart D, Lok S, Martin DW, Roybal KT, Kim KC. convertibleCARs: A chimeric antigen receptor system for flexible control of activity and antigen targeting. Commun Biol. 2020;3(1):296. 32518350 10.1038/s42003-020-1021-2 PMC7283332

[146] Kembuan GJ, Kim JY, Maus MV, Jan M. Targeting solid tumor antigens with chimeric receptors: Cancer biology meets synthetic immunology. Trends Cancer. 2024;10(4):312–331. 38355356 10.1016/j.trecan.2024.01.003 PMC11006585

[147] Van den Eynde A, Gehrcken L, Verhezen T, Lau HW, Hermans C, Lambrechts H, Flieswasser T, Quatannens D, Roex G, Zwaenepoel K, et al. IL-15-secreting CAR natural killer cells directed toward the pan-cancer target CD70 eliminate both cancer cells and cancer-associated fibroblasts. J Hematol Oncol. 2024;17(1):8. 38331849 10.1186/s13045-024-01525-w PMC10854128

[148] Daher M, Basar R, Gokdemir E, Baran N, Uprety N, Nunez Cortes AK, Mendt M, Kerbauy LN, Banerjee PP, Shanley M, et al. Targeting a cytokine checkpoint enhances the fitness of armored cord blood CAR-NK cells. Blood. 2021;137(5):624–636. 32902645 10.1182/blood.2020007748 PMC7869185

[149] Gurney M, Kundu S, Pandey S, O’Dwyer M. Feeder cells at the interface of natural killer cell activation, expansion and gene editing. Front Immunol. 2022;13: Article 802906. 35222382 10.3389/fimmu.2022.802906 PMC8873083

[150] Denman CJ, Senyukov VV, Somanchi SS, Phatarpekar PV, Kopp LM, Johnson JL, Singh H, Hurton L, Maiti SN, Huls MH, et al. Membrane-bound IL-21 promotes sustained ex vivo proliferation of human natural killer cells. PLOS ONE. 2012;7(1): Article e30264. 22279576 10.1371/journal.pone.0030264 PMC3261192

[151] Foltz JA, Hess BT, Bachanova V, Bartlett NL, Berrien-Elliott MM, McClain E, Becker-Hapak M, Foster M, Schappe T, Kahl B, et al. Phase I trial of N-803, an IL15 receptor agonist, with rituximab in patients with indolent non-Hodgkin lymphoma. Clin Cancer Res. 2021;27(12):3339–3350. 33832946 10.1158/1078-0432.CCR-20-4575 PMC8197753

[152] Romee R, Cooley S, Berrien-Elliott MM, Westervelt P, Verneris MR, Wagner JE, Weisdorf DJ, Blazar BR, Ustun C, DeFor TE, et al. First-in-human phase 1 clinical study of the IL-15 superagonist complex ALT-803 to treat relapse after transplantation. Blood. 2018;131(23):2515–2527. 29463563 10.1182/blood-2017-12-823757 PMC5992862

[153] Romee R, Rosario M, Berrien-Elliott MM, Wagner JA, Jewell BA, Schappe T, Leong JW, Abdel-Latif S, Schneider SE, Willey S, et al. Cytokine-induced memory-like natural killer cells exhibit enhanced responses against myeloid leukemia. Sci Transl Med. 2016;8(357): Article 357ra123. 27655849 10.1126/scitranslmed.aaf2341 PMC5436500

[154] Romee R, Schneider SE, Leong JW, Chase JM, Keppel CR, Sullivan RP, Cooper MA, Fehniger TA. Cytokine activation induces human memory-like NK cells. Blood. 2012;120(24):4751–4760. 22983442 10.1182/blood-2012-04-419283 PMC3520618

[155] Gang M, Marin ND, Wong P, Neal CC, Marsala L, Foster M, Schappe T, Meng W, Tran J, Schaettler M, et al. CAR-modified memory-like NK cells exhibit potent responses to NK-resistant lymphomas. Blood. 2020;136(20):2308–2318. 32614951 10.1182/blood.2020006619 PMC7702478

[156] Dong H, Ham JD, Hu G, Xie G, Vergara J, Liang Y, Ali A, Tarannum M, Donner H, Baginska J, et al. Memory-like NK cells armed with a neoepitope-specific CAR exhibit potent activity against NPM1 mutated acute myeloid leukemia. Proc Natl Acad Sci USA. 2022;119(25): Article e2122379119. 35696582 10.1073/pnas.2122379119 PMC9231490

[157] Tarannum M, Dinh K, Vergara J, Birch G, Abdulhamid YZ, Kaplan IE, et al. CAR memory-like NK cells targeting the membrane proximal domain of mesothelin demonstrate promising activity in ovarian cancer. Sci Adv. 2024;10(28): Article eadn0881. 38996027 10.1126/sciadv.adn0881 PMC11244547

[158] Carrington EM, Zhan Y, Brady JL, Zhang JG, Sutherland RM, Anstee NS, Schenk RL, Vikstrom IB, Delconte RB, Segal D, et al. Anti-apoptotic proteins BCL-2, MCL-1 and A1 summate collectively to maintain survival of immune cell populations both in vitro and in vivo. Cell Death Differ. 2017;24(5):878–888. 28362427 10.1038/cdd.2017.30 PMC5423112

[159] Delconte RB, Guittard G, Goh W, Hediyeh-Zadeh S, Hennessy RJ, Rautela J, Davis MJ, Souza-Fonseca-Guimaraes F, Nunès JA, Huntington ND. NK cell priming from endogenous homeostatic signals is modulated by CIS. Front Immunol. 2020;11:75. 32082327 10.3389/fimmu.2020.00075 PMC7005222

[160] Beavis PA, Divisekera U, Paget C, Chow MT, John LB, Devaud C, Dwyer K, Stagg J, Smyth MJ, Darcy PK. Blockade of A_2A_ receptors potently suppresses the metastasis of CD73^+^ tumors. Proc Natl Acad Sci USA. 2013;110(36):14711–14716. 23964122 10.1073/pnas.1308209110 PMC3767556

[161] Wang W, Liu Y, He Z, Li L, Liu S, Jiang M, Zhao B, Deng M, Wang W, Mi X, et al. Breakthrough of solid tumor treatment: CAR-NK immunotherapy. Cell Death Discov. 2024;10(1):40. 38245520 10.1038/s41420-024-01815-9 PMC10799930

[162] Miao L, Lu C, Zhang B, Li H, Zhao X, Chen H, Liu Y, Cui X. Advances in metabolic reprogramming of NK cells in the tumor microenvironment on the impact of NK therapy. J Transl Med. 2024;22(1):229. 38433193 10.1186/s12967-024-05033-w PMC10909296

[163] Zhong Y, Liu J. Emerging roles of CAR-NK cell therapies in tumor immunotherapy: Current status and future directions. Cell Death Discov. 2024;10(1):318. 38987565 10.1038/s41420-024-02077-1 PMC11236993

[164] Arias J, Yu J, Varshney M, Inzunza J, Nalvarte I. Hematopoietic stem cell- and induced pluripotent stem cell-derived CAR-NK cells as reliable cell-based therapy solutions. Stem Cells Transl Med. 2021;10(7):987–995. 33634954 10.1002/sctm.20-0459 PMC8235144

[165] Jørgensen LV, Christensen EB, Barnkob MB, Barington T. The clinical landscape of CAR NK cells. Exp Hematol Oncol. 2025;14(1):46. 40149002 10.1186/s40164-025-00633-8 PMC11951618

[166] Di Stasi A, Tey SK, Dotti G, Fujita Y, Kennedy-Nasser A, Martinez C, Straathof K, Liu E, Durett AG, Grilley B, et al. Inducible apoptosis as a safety switch for adoptive cell therapy. N Engl J Med. 2011;365(18):1673–1683. 22047558 10.1056/NEJMoa1106152 PMC3236370

[167] Liu E, Tong Y, Dotti G, Shaim H, Savoldo B, Mukherjee M, Orange J, Wan X, Lu X, Reynolds A, et al. Cord blood NK cells engineered to express IL-15 and a CD19-targeted CAR show long-term persistence and potent antitumor activity. Leukemia. 2018;32(2):520–531. 28725044 10.1038/leu.2017.226 PMC6063081

[168] Garrison BS, Deng H, Yucel G, Frankel NW, Guzman-Ayala M, Gordley R, Hung M, Lee D, Gainer M, Loving K, et al. FLT3 OR CD33 NOT EMCN logic gated CAR-NK cell therapy (SENTI-202) for precise targeting of AML. Blood. 2021;138(Suppl 1):2799.34724566

[169] Restifo NP, Dudley ME, Rosenberg SA. Adoptive immunotherapy for cancer: Harnessing the T cell response. Nat Rev Immunol. 2012;12(4):269–281. 22437939 10.1038/nri3191 PMC6292222

[170] Shimasaki N, Jain A, Campana D. NK cells for cancer immunotherapy. Nat Rev Drug Discov. 2020;19(3):200–218. 31907401 10.1038/s41573-019-0052-1

[171] Gattinoni L, Finkelstein SE, Klebanoff CA, Antony PA, Palmer DC, Spiess PJ, Hwang LN, Yu Z, Wrzesinski C, Heimann DM, et al. Removal of homeostatic cytokine sinks by lymphodepletion enhances the efficacy of adoptively transferred tumor-specific CD8^+^ T cells. J Exp Med. 2005;202(7):907–912. 16203864 10.1084/jem.20050732 PMC1397916

[172] Soriani A, Fionda C, Ricci B, Iannitto ML, Cippitelli M, Santoni A. Chemotherapy-elicited upregulation of NKG2D and DNAM-1 ligands as a therapeutic target in multiple myeloma. Onco Targets Ther. 2013;2(12): Article e26663. 10.4161/onci.26663 PMC3912005 24498552

[173] Zhai J, Gu X, Liu Y, Hu Y, Jiang Y, Zhang Z. Chemotherapeutic and targeted drugs-induced immunogenic cell death in cancer models and antitumor therapy: An update review. Front Pharmacol. 2023;14:1152934. 37153795 10.3389/fphar.2023.1152934 PMC10160433

[174] Vanmeerbeek I, Sprooten J, De Ruysscher D, Tejpar S, Vandenberghe P, Fucikova J, Spisek R, Zitvogel L, Kroemer G, Galluzzi L, et al. Trial watch: Chemotherapy-induced immunogenic cell death in immuno-oncology. Onco Targets Ther. 2020;9(1):1703449. 10.1080/2162402X.2019.1703449 PMC6959434 32002302

[175] Yu M, Han J, Cui P, Dai M, Li H, Zhang J, Xiu R. Cisplatin up-regulates ICAM-1 expression in endothelial cell via a NF-kappaB dependent pathway. Cancer Sci. 2008;99(2):391–397. 18271937 10.1111/j.1349-7006.2008.00696.x PMC11159323

[176] Frese KK, Neesse A, Cook N, Bapiro TE, Lolkema MP, Jodrell DI, Tuveson DA. nab-Paclitaxel potentiates gemcitabine activity by reducing cytidine deaminase levels in a mouse model of pancreatic cancer. Cancer Discov. 2012;2(3):260–269. 22585996 10.1158/2159-8290.CD-11-0242 PMC4866937

[177] Vincent J, Mignot G, Chalmin F, Ladoire S, Bruchard M, Chevriaux A, Martin F, Apetoh L, Rébé C, Ghiringhelli F. 5-Fluorouracil selectively kills tumor-associated myeloid-derived suppressor cells resulting in enhanced T cell-dependent antitumor immunity. Cancer Res. 2010;70(8):3052–3061. 20388795 10.1158/0008-5472.CAN-09-3690

[178] Klapdor R, Wang S, Hacker U, Büning H, Morgan M, Dörk T, Hillemanns P, Schambach A. Improved killing of ovarian cancer stem cells by combining a novel chimeric antigen receptor-based immunotherapy and chemotherapy. Hum Gene Ther. 2017;28(10):886–896. 28836469 10.1089/hum.2017.168

[179] Klapdor R, Wang S, Morgan MA, Zimmermann K, Hachenberg J, Buening H, Doerk T, Hillemanns P, Schambach A. NK cell-mediated eradication of ovarian cancer cells with a novel chimeric antigen receptor directed against CD44. Biomedicine. 2021;9(10): Article 1339. 10.3390/biomedicines9101339 PMC8533227 34680456

[180] Siegler EL, Kim YJ, Chen X, Siriwon N, Mac J, Rohrs JA, Bryson PD, Wang P. Combination cancer therapy using chimeric antigen receptor-engineered natural killer cells as drug carriers. Mol Ther. 2017;25(12):2607–2619. 28919377 10.1016/j.ymthe.2017.08.010 PMC5768663

[181] Huang S, Xing F, Dai Y, Zhang Z, Zhou G, Yang S, Liu YC, Yuan Z, Luo KQ, Ying T, et al. Navigating chimeric antigen receptor-engineered natural killer cells as drug carriers via three-dimensional mapping of the tumor microenvironment. J Control Release. 2023;362:524–535. 37673307 10.1016/j.jconrel.2023.09.007

[182] Zhu Y, An X, Zhang X, Qiao Y, Zheng T, Li X. STING: A master regulator in the cancer-immunity cycle. Mol Cancer. 2019;18(1):152. 31679519 10.1186/s12943-019-1087-y PMC6827255

[183] Deng L, Liang H, Xu M, Yang X, Burnette B, Arina A, Li XD, Mauceri H, Beckett M, Darga T, et al. STING-dependent cytosolic DNA sensing promotes radiation-induced type I interferon-dependent antitumor immunity in immunogenic tumors. Immunity. 2014;41(5):843–852. 25517616 10.1016/j.immuni.2014.10.019 PMC5155593

[184] Guo S, Yao Y, Tang Y, Xin Z, Wu D, Ni C, Huang J, Wei Q, Zhang T. Radiation-induced tumor immune microenvironments and potential targets for combination therapy. Signal Transduct Target Ther. 2023;8(1):205. 37208386 10.1038/s41392-023-01462-z PMC10199044

[185] Barker HE, Paget JTE, Khan AA, Harrington KJ. The tumour microenvironment after radiotherapy: Mechanisms of resistance and recurrence. Nat Rev Cancer. 2015;15(7):409–425. 26105538 10.1038/nrc3958 PMC4896389

[186] Hovhannisyan L, Riether C, Aebersold DM, Medová M, Zimmer Y. CAR T cell-based immunotherapy and radiation therapy: Potential, promises and risks. Mol Cancer. 2023;22(1):82. 37173782 10.1186/s12943-023-01775-1 PMC10176707

[187] Akmansu M, Unsal D, Bora H, Elbeg S. Influence of locoregional radiation treatment on tumor necrosis factor-alpha and interleukin-6 in the serum of patients with head and neck cancer. Cytokine. 2005;31(1):41–45. 15878671 10.1016/j.cyto.2005.02.009

[188] Gerber SA, Sedlacek AL, Cron KR, Murphy SP, Frelinger JG, Lord EM. IFN-γ mediates the antitumor effects of radiation therapy in a murine colon tumor. Am J Pathol. 2013;182(6):2345–2354. 23583648 10.1016/j.ajpath.2013.02.041 PMC3668027

[189] Gasser S, Orsulic S, Brown EJ, Raulet DH. The DNA damage pathway regulates innate immune system ligands of the NKG2D receptor. Nature. 2005;436(7054):1186–1190. 15995699 10.1038/nature03884 PMC1352168

[190] Gasser S, Raulet DH. Activation and self-tolerance of natural killer cells. Immunol Rev. 2006;214:130–142. 17100881 10.1111/j.1600-065X.2006.00460.x

[191] Wei SC, Duffy CR, Allison JP. Fundamental mechanisms of immune checkpoint blockade therapy. Cancer Discov. 2018;8(9):1069–1086. 30115704 10.1158/2159-8290.CD-18-0367

[192] Concha-Benavente F, Kansy B, Moskovitz J, Moy J, Chandran U, Ferris RL. PD-L1 mediates dysfunction in activated PD-1+ NK cells in head and neck cancer patients. Cancer Immunol Res. 2018;6(12):1548–1560. 30282672 10.1158/2326-6066.CIR-18-0062 PMC6512340

[193] Oyer JL, Gitto SB, Altomare DA, Copik AJ. PD-L1 blockade enhances anti-tumor efficacy of NK cells. Onco Targets Ther. 2018;7(11): Article e1509819. 10.1080/2162402X.2018.1509819 PMC6205063 30377572

[194] Judge SJ, Dunai C, Aguilar EG, Vick SC, Sturgill IR, Khuat LT, Stoffel KM, Van Dyke J, Longo DL, Darrow MA, et al. Minimal PD-1 expression in mouse and human NK cells under diverse conditions. J Clin Invest. 2020;130(6):3051–3068. 32134744 10.1172/JCI133353 PMC7260004

[195] Liu F, Tarannum M, Zhao Y, Zhang YJ, Ham JD, Lei K, Qiang Y, Deng X, Nguyen M, Dinh K, et al. Selective HLA knockdown and PD-L1 expression prevent allogeneic CAR-NK cell rejection and enhance safety and anti-tumor responses in xenograft mice. Nat Commun. 2025;16(1):8809. 41062480 10.1038/s41467-025-63863-8 PMC12508061

[196] Liu WN, So WY, Harden SL, Fong SY, Wong MX, Tan WW, Tan SY, Ong JK, Rajarethinam R, Liu M, et al. Successful targeting of PD-1/PD-L1 with chimeric antigen receptor-natural killer cells and nivolumab in a humanized mouse cancer model. Sci Adv. 2022;8(47): Article eadd1187. 36417514 10.1126/sciadv.add1187 PMC9683725

[197] Li J, Hu H, Lian H, Yang S, Liu M, He J, Cao B, Chen D, Hu Y, Zhi C, et al. NK-92MI cells engineered with anti-claudin-6 chimeric antigen receptors in immunotherapy for ovarian cancer. Int J Biol Sci. 2024;20(5):1578–1601. 38481806 10.7150/ijbs.88539 PMC10929190

[198] Strassheimer F, Elleringmann P, Ludmirski G, Roller B, Macas J, Alekseeva T, Cakmak P, Aliraj B, Krenzlin H, Demes MC, et al. CAR-NK cell therapy combined with checkpoint inhibition induces an NKT cell response in glioblastoma. Br J Cancer. 2025;132(9):849–860. 40102596 10.1038/s41416-025-02977-8 PMC12041480

[199] Burger MC, Forster MT, Romanski A, Straßheimer F, Macas J, Zeiner PS, Steidl E, Herkt S, Weber KJ, Schupp J, et al. Intracranial injection of natural killer cells engineered with a HER2-targeted chimeric antigen receptor in patients with recurrent glioblastoma. Neuro-Oncology. 2023;25(11):2058–2071. 37148198 10.1093/neuonc/noad087 PMC10628939

[200] Navin I, Dysthe M, Menon PS, Baumgartner C, Sauer T, Varadarajan N, Parihar R. TIGIT affects CAR NK cell effector function in the solid tumor microenvironment by modulating immune synapse strength. Cancer Immunol Res. 2025;13(10):1576–1590. 40736004 10.1158/2326-6066.CIR-24-0919 PMC12453546

[201] Gallois A, Silva I, Osman I, Bhardwaj N. Reversal of natural killer cell exhaustion by TIM-3 blockade. Onco Targets Ther. 2014;3(12): Article e946365. 10.4161/21624011.2014.946365 PMC4353130 25964857

[202] Cai L, Li Y, Tan J, Xu L, Li Y. Targeting LAG-3, TIM-3, and TIGIT for cancer immunotherapy. J Hematol Oncol. 2023;16(1):101. 37670328 10.1186/s13045-023-01499-1 PMC10478462

[203] Alderson KL, Sondel PM. Clinical cancer therapy by NK cells via antibody-dependent cell-mediated cytotoxicity. J Biomed Biotechnol. 2011;2011(1): Article 379123. 21660134 10.1155/2011/379123 PMC3110303

[204] Romee R, Foley B, Lenvik T, Wang Y, Zhang B, Ankarlo D, Luo X, Cooley S, Verneris M, Walcheck B, et al. NK cell CD16 surface expression and function is regulated by a disintegrin and metalloprotease-17 (ADAM17). Blood. 2013;121(18):3599–3608. 23487023 10.1182/blood-2012-04-425397 PMC3643761

[205] Ghobadi A, Bachanova V, Patel K, Park JH, Flinn I, Riedell PA, Bachier C, Diefenbach CS, Wong C, Bickers C, et al. Induced pluripotent stem-cell-derived CD19-directed chimeric antigen receptor natural killer cells in B-cell lymphoma: A phase 1, first-in-human trial. Lancet. 2025;405(10473):127–136. 39798981 10.1016/S0140-6736(24)02462-0 PMC11827677

[206] Tsao LC, Wang JS, Ma X, Sodhi S, Ragusa JV, Liu B, McBane J, Wang T, Wei J, Liu CX, et al. Effective extracellular payload release and immunomodulatory interactions govern the therapeutic effect of trastuzumab deruxtecan (T-DXd). Nat Commun. 2025;16(1):3167. 40175391 10.1038/s41467-025-58266-8 PMC11965298

[207] Montes de Oca R, Alavi AS, Vitali N, Bhattacharya S, Blackwell C, Patel K, Seestaller-Wehr L, Kaczynski H, Shi H, Dobrzynski E, et al. Belantamab Mafodotin (GSK2857916) drives immunogenic cell death and immune-mediated antitumor responses in vivo. Mol Cancer Ther. 2021;20(10):1941–1955. 34253590 10.1158/1535-7163.MCT-21-0035 PMC9398105

[208] Ruffo E, Parikh A, Falcone G, Ma Y, Deiters A, Lohmueller J. 313 antibody-drug conjugates (ADCs) as adaptors for universal CAR T cells. J ImmunoTher Cancer. 2024;12(2).

[209] Ruffo E, Butchy AA, Tivon Y, So V, Kvorjak M, Parikh A, Adams EL, Miskov-Zivanov N, Finn OJ, Deiters A, et al. Post-translational covalent assembly of CAR and synNotch receptors for programmable antigen targeting. Nat Commun. 2023;14(1):2463. 37160880 10.1038/s41467-023-37863-5 PMC10169838

[210] Vallera DA, Felices M, McElmurry R, McCullar V, Zhou X, Schmohl JU, Zhang B, Lenvik AJ, Panoskaltsis-Mortari A, Verneris MR, et al. IL15 trispecific killer engagers (TriKE) make natural killer cells specific to CD33+ targets while also inducing persistence, in vivo expansion, and enhanced function. Clin Cancer Res. 2016;22(14):3440–3450. 26847056 10.1158/1078-0432.CCR-15-2710 PMC4947440

[211] Miller JS, Warlick ED, Wangen R, Zorko N, Hinderlie P, Lewis D, Vallera DA, Felices M. 965MO GTB-3550 tri-specific killer engager safely activates and delivers IL-15 to NK cells, but not T-cells, in immune suppressed patients with advanced myeloid malignancies, a novel paradigm exportable to solid tumors expressing Her2 or B7H3. Ann Oncol. 2021;32:S834.

[212] Khaw MJ, Zorko NA, Kennedy PR, Bendzick LE, Shackelford M, Selleck C, Hinderlie P, Walker JT, Soignier Y, Lyons RC, et al. Novel trispecific killer engager targeting B7-H3 enhances natural killer cell antitumor activity against head and neck cancer. J ImmunoTher Cancer. 2025;13(7): Article e011370. 40707133 10.1136/jitc-2024-011370 PMC12306285

[213] Zhang Q, Zhang H, Ding J, Liu H, Li H, Li H, Lu M, Miao Y, Li L, Zheng J. Combination therapy with EpCAM-CAR-NK-92 cells and regorafenib against human colorectal cancer models. J Immunol Res. 2018;2018(1):4263520. 30410941 10.1155/2018/4263520 PMC6205314

[214] Zhang Q, Tian K, Xu J, Zhang H, Li L, Fu Q, Chai D, Li H, Zheng J. Synergistic effects of cabozantinib and EGFR-specific CAR-NK-92 cells in renal cell carcinoma. J Immunol Res. 2017;2017(1):6915912. 29423418 10.1155/2017/6915912 PMC5750507

[215] Sedloev D, Chen Q, Unglaub JM, Schanda N, Hao Y, Besiridou E, Neuber B, Schmitt A, Raffel S, Liu Y, et al. Proteasome inhibition enhances the anti-leukemic efficacy of chimeric antigen receptor (CAR) expressing NK cells against acute myeloid leukemia. J Hematol Oncol. 2024;17(1):85. 39285441 10.1186/s13045-024-01604-y PMC11406742

[216] Jo DH, Kaczmarek S, Khan AU, Pervin J, Clark DM, Gadde S, Wang L, McComb S, Visram A, Lee SH. Entinostat, a histone deacetylase inhibitor, enhances CAR-NK cell anti-tumor activity by sustaining CAR expression. Front Immunol. 2025;16:1533044. 40124378 10.3389/fimmu.2025.1533044 PMC11925867

[217] Fukumura D, Kloepper J, Amoozgar Z, Duda DG, Jain RK. Enhancing cancer immunotherapy using antiangiogenics: Opportunities and challenges. Nat Rev Clin Oncol. 2018;15(5):325–340. 29508855 10.1038/nrclinonc.2018.29 PMC5921900

[218] Draghiciu O, Nijman HW, Hoogeboom BN, Meijerhof T, Daemen T. Sunitinib depletes myeloid-derived suppressor cells and synergizes with a cancer vaccine to enhance antigen-specific immune responses and tumor eradication. Onco Targets Ther. 2015;4(3): Article e989764. 10.4161/2162402X.2014.989764 PMC4404834 25949902

[219] Bhat J, Dubin S, Dananberg A, Quabius ES, Fritsch J, Dowds CM, Saxena A, Chitadze G, Lettau M, Kabelitz D. Histone deacetylase inhibitor modulates NKG2D receptor expression and memory phenotype of human gamma/delta T cells upon interaction with tumor cells. Front Immunol. 2019;10:569. 30972064 10.3389/fimmu.2019.00569 PMC6445873

[220] Lu L, Yang C, Zhou X, Wu L, Hong X, Li W, Wang X, Yang Y, Cao D, Zhang A, et al. STING signaling promotes NK cell antitumor immunity and maintains a reservoir of TCF-1+ NK cells. Cell Rep. 2023;42(9): Article 113108. 37708030 10.1016/j.celrep.2023.113108

[221] Liu G-H, Chen T, Zhang X, Ma X-L, Shi H-S. Small molecule inhibitors targeting the cancers. MedComm. 2020;3(4): Article e181. 10.1002/mco2.181 PMC9560750 36254250

[222] Xu L, Sun H, Lemoine NR, Xuan Y, Wang P. Oncolytic vaccinia virus and cancer immunotherapy. Front Immunol. 2023;14:1324744. 38283361 10.3389/fimmu.2023.1324744 PMC10811104

[223] Ma R, Lu T, Li Z, Teng KY, Mansour AG, Yu M, Tian L, Xu B, Ma S, Zhang J, et al. An oncolytic virus expressing IL15/IL15Rα combined with off-the-shelf EGFR-CAR NK cells targets glioblastoma. Cancer Res. 2021;81(13):3635–3648. 34006525 10.1158/0008-5472.CAN-21-0035 PMC8562586

[224] Chu Y, Tian M, Saini U, Ayala-Cuesta J, Klose K, Mendelowitz AS, Foley K, Ozkaynak MF, Luo W, Cripe TP, et al. Combinatorial immunotherapy with anti-ROR1 CAR NK cells and an IL-21 secreting oncolytic virus against neuroblastoma. Mol Ther Oncol. 2025;33(1): Article 200927. 39895691 10.1016/j.omton.2024.200927 PMC11783442

[225] Biegert G, Shaw AR, Morita D, Porter C, Matsumoto R, Jatta L, Crooks N, Woods M, Yao QC, Parihar R, et al. Oncolytic adeno-immunotherapy improves allogeneic adoptive HER2.CAR-NK function against pancreatic ductal adenocarcinoma. Mol Ther Oncol. 2025;33(2): Article 201006. 40546314 10.1016/j.omton.2025.201006 PMC12179663

[226] Wang X, Wei G, Karki KB, Chan W, Viskovska M, Williams A, Chang N, Jiang H. Developing a novel combination therapy using engineered chimeric antigen receptor natural killer cells targeting avsialidase with avsialidase-armed oncolytic vaccinia virus in solid tumor models. Cancer Res. 2022;82(Suppl 12):6225.

[227] Kong R, Liu B, Wang H, Lu T, Zhou X. CAR-NK cell therapy: Latest updates from the 2024 ASH annual meeting. J Hematol Oncol. 2025;18(1):22. 40025557 10.1186/s13045-025-01677-3 PMC11872314

[228] Li J, Chen P, Ma W. The next frontier in immunotherapy: Potential and challenges of CAR-macrophages. Exp Hematol Oncol. 2024;13(1):76. 39103972 10.1186/s40164-024-00549-9 PMC11302330

[229] Wang M, Qin Z, Bian XW, Shi Y. Harnessing chimeric antigen receptor macrophages against solid tumors. Cancer Commun. 2025;45(11):1344–1366. 10.1002/cac2.70053 PMC12629864 40820270

[230] Li G, Wu X, Chan IH, Trager JB. A combination of CAR-NK and CAR-T cells results in rapid and persistent anti-tumor efficacy while reducing CAR-T cell mediated cytokine release and T-cell proliferation. Cancer Res. 2020;80(Suppl 16):4235.

[231] Klichinsky M, Ruella M, Shestova O, Lu XM, Best A, Zeeman M, Schmierer M, Gabrusiewicz K, Anderson NR, Petty NE, et al. Human chimeric antigen receptor macrophages for cancer immunotherapy. Nat Biotechnol. 2020;38(8):947–953. 32361713 10.1038/s41587-020-0462-y PMC7883632

[232] Henze A-T, Mazzone M. The impact of hypoxia on tumor-associated macrophages. J Clin Invest. 2016;126(10):3672–3679. 27482883 10.1172/JCI84427 PMC5096805

[233] Lu J, Ma Y, Li Q, Xu Y, Xue Y, Xu S. CAR macrophages: A promising novel immunotherapy for solid tumors and beyond. Biomark Res. 2024;12(1):86. 39175095 10.1186/s40364-024-00637-2 PMC11342599

[234] Reiss KA, Angelos MG, Dees EC, Yuan Y, Ueno NT, Pohlmann PR, Johnson ML, Chao J, Shestova O, Serody JS, et al. CAR-macrophage therapy for HER2-overexpressing advanced solid tumors: A phase 1 trial. Nat Med. 2025;31(4):1171–1182. 39920391 10.1038/s41591-025-03495-z

[235] de Picciotto S, DeVita N, Hsiao CJ, Honan C, Tse SW, Nguyen M, Ferrari JD, Zheng W, Wipke BT, Huang E. Selective activation and expansion of regulatory T cells using lipid encapsulated mRNA encoding a long-acting IL-2 mutein. Nat Commun. 2022;13(1):3866. 35790728 10.1038/s41467-022-31130-9 PMC9256694

[236] Raeber ME, Sahin D, Karakus U, Boyman O. A systematic review of interleukin-2-based immunotherapies in clinical trials for cancer and autoimmune diseases. EBioMedicine. 2023;90: Article 104539. 37004361 10.1016/j.ebiom.2023.104539 PMC10111960

[237] Waldmann TA. The shared and contrasting roles of IL2 and IL15 in the life and death of normal and neoplastic lymphocytes: Implications for cancer therapy. Cancer Immunol Res. 2015;3(3):219–227. 25736261 10.1158/2326-6066.CIR-15-0009 PMC4351780

[238] Berrien-Elliott MM, Becker-Hapak M, Cashen AF, Jacobs M, Wong P, Foster M, McClain E, Desai S, Pence P, Cooley S, et al. Systemic IL-15 promotes allogeneic cell rejection in patients treated with natural killer cell adoptive therapy. Blood. 2022;139(8):1177–1183. 34797911 10.1182/blood.2021011532 PMC9211446

[239] Imamura M, Shook D, Kamiya T, Shimasaki N, Chai SM, Coustan-Smith E, Imai C, Campana D. Autonomous growth and increased cytotoxicity of natural killer cells expressing membrane-bound interleukin-15. Blood. 2014;124(7):1081–1088. 25006133 10.1182/blood-2014-02-556837

[240] Le J, Dian Y, Zhao D, Guo Z, Luo Z, Chen X, Zeng F, Deng G. Single-cell multi-omics in cancer immunotherapy: From tumor heterogeneity to personalized precision treatment. Mol Cancer. 2025;24(1):221. 40855431 10.1186/s12943-025-02426-3 PMC12376342

[241] Uhlén M, Fagerberg L, Hallström BM, Lindskog C, Oksvold P, Mardinoglu A, Sivertsson Å, Kampf C, Sjöstedt E, Asplund A, et al. Proteomics. Tissue-based map of the human proteome. Science. 2015;347(6220): Article 1260419. 25613900 10.1126/science.1260419

[242] Chang TD, Chen YJ, Luo JL, Zhang C, Chen SY, Lin ZQ, Zhang PD, Shen YX, Tang TX, Li H, et al. Adaptation of natural killer cells to hypoxia: A review of the transcriptional, translational, and metabolic processes. Immunotargets Ther. 2025;99–121. 39990274 10.2147/ITT.S492334 PMC11846490

[243] Ghosh S, Dutta R, Goswami D, Ghatak D, De R. Mitochondrial dynamics and metabolic attributes regulate function of natural killer cell and infiltration in tumor microenvironment modulating disease progression. Biochim Biophys Acta Rev Cancer. 2025;1880(6): Article 189471. 41075850 10.1016/j.bbcan.2025.189471

[244] Verhezen T, Wouters A, Smits E, De Waele J. Powering immunity: Mitochondrial dynamics in natural killer cells. Trends Mol Med. 2025. 10.1016/j.molmed.2025.04.004 40393875

[245] Assmann N, O’Brien KL, Donnelly RP, Dyck L, Zaiatz-Bittencourt V, Loftus RM, Heinrich P, Oefner PJ, Lynch L, Gardiner CM, et al. Srebp-controlled glucose metabolism is essential for NK cell functional responses. Nat Immunol. 2017;18(11):1197–1206. 28920951 10.1038/ni.3838

[246] Zhao R, He B, Huang L, Wu Y, Liu T, Liu J, Zhao M, Zhong T, Zhang Y, Zhang X, et al. Targeting AQP5-mediated arginine deprivation in gastric cancer stem cells restores NK cell anti-tumor immunity. Cell Rep Med. 2025;6(9): Article 102333. 40961922 10.1016/j.xcrm.2025.102333 PMC12490235

[247] Llibre A, Kucuk S, Gope A, Certo M, Mauro C. Lactate: A key regulator of the immune response. Immunity. 2025;58(3):535–554. 40073846 10.1016/j.immuni.2025.02.008

[248] Rodríguez-Pampín I, González-Pico L, Selas A, Andújar A, Prieto-Díaz R, Sotelo E. Targeting the adenosinergic axis in cancer immunotherapy: Insights into A2A and A2B receptors and novel clinical combination strategies. Pharmacol Rev. 2025;77(6): Article 100092. 41101027 10.1016/j.pharmr.2025.100092

[249] Lei W, Zhou K, Lei Y, Li Q, Zhu H. Gut microbiota shapes cancer immunotherapy responses. NPJ Biofilms Microbiomes. 2025;11(1):143. 40715107 10.1038/s41522-025-00786-8 PMC12297587

[250] Smith M, Dai A, Ghilardi G, Amelsberg KV, Devlin SM, Pajarillo R, Slingerland JB, Beghi S, Herrera PS, Giardina P, et al. Gut microbiome correlates of response and toxicity following anti-CD19 CAR T cell therapy. Nat Med. 2022;28(4):713–723. 35288695 10.1038/s41591-022-01702-9 PMC9434490

[251] Carlini F, Squillario M, Casella V, Capaia M, Lusi V, Bagnara D, Colombo M, Palmeri S, Ivaldi F, Loiacono F, et al. Butyrate enhances CD56bright NK cell-driven killing of activated T cells and modulates NK cell chromatin accessibility. Genes Immun. 2025;26(4):342–351. 40506519 10.1038/s41435-025-00338-2

[252] Yang W, Cong Y. Gut microbiota-derived metabolites in the regulation of host immune responses and immune-related inflammatory diseases. Cell Mol Immunol. 2021;18(4):866–877. 33707689 10.1038/s41423-021-00661-4 PMC8115644

[253] Xie J, Liu M, Deng X, Tang Y, Zheng S, Ou X, Tang H, Xie X, Wu M, Zou Y. Gut microbiota reshapes cancer immunotherapy efficacy: Mechanisms and therapeutic strategies. iMeta. 2024;3(1): Article e156. 38868510 10.1002/imt2.156 PMC10989143

[254] Berbudi A, Khairani S, Tjahjadi AI. Interplay between insulin resistance and immune dysregulation in type 2 diabetes mellitus: Implications for therapeutic interventions. Immunotargets Ther. 2025;14:359–382. 40196377 10.2147/ITT.S499605 PMC11974557

[255] Lutz CT, Quinn LS. Sarcopenia, obesity, and natural killer cell immune senescence in aging: Altered cytokine levels as a common mechanism. Aging. 2012;4(8):535–546. 22935594 10.18632/aging.100482 PMC3461341

[256] Liu Z, Liang Q, Ren Y, Guo C, Ge X, Wang L, Cheng Q, Luo P, Zhang Y, Han X. Immunosenescence: Molecular mechanisms and diseases. Signal Transduct Target Ther. 2023;8(1):200. 37179335 10.1038/s41392-023-01451-2 PMC10182360

[257] Nguyen TT, Ho P, Staudt S, Gregoire C, Ziegler-Martin K, Jassin M, Block A, Hudecek M, Melenhorst JJ, Caers J, et al. Fine tuning towards the next generation of engineered T cells. Nat Biomed Eng. 2025;9(10):1610–1631. 41073806 10.1038/s41551-025-01492-8

[258] Biederstädt A, Rezvani K. Engineered natural killer cells for cancer therapy. Cancer Cell. 2025;43(11):1987–2013. 41135520 10.1016/j.ccell.2025.09.013 PMC12631954

[259] Odeh‐Couvertier VY, Dwarshuis NJ, Colonna MB, Levine BL, Edison AS, Kotanchek T, Roy K, Torres‐Garcia W. Predicting T-cell quality during manufacturing through an artificial intelligence-based integrative multiomics analytical platform. Bioeng Transl Med. 2022;7(2): Article e10282. 35600660 10.1002/btm2.10282 PMC9115702

[260] Nikolic I, Cursons J, Shields B, Chappaz S, Sudholz H, Meng X, Constantinescu P, Vijayakumaran R, D’Angelo M, Foroutan M, et al. Enhancing anti-tumor immunity of natural killer cells through targeting IL-15R signaling. Cancer Cell. 2025;43(11):2034–2050. 40513576 10.1016/j.ccell.2025.05.011

[261] Biederstädt A, Basar R, Park JM, Uprety N, Shrestha R, Silva FR, Dede M, Watts J, Acharya S, Xiong D, et al. Genome-wide CRISPR screens identify critical targets to enhance CAR-NK cell antitumor potency. Cancer Cell. 2025;43(11):2069–2088. 40845844 10.1016/j.ccell.2025.07.021 PMC12396527

[262] Wang J, Tao X, Zhu J, Dai Z, Du Y, Xie Y, Chu X, Fu G, Lei Z. Tumor organoid-immune co-culture models: Exploring a new perspective of tumor immunity. Cell Death Discov. 2025;11(1):195. 40268893 10.1038/s41420-025-02407-x PMC12019369

[263] Ma C, Wang H, Liu L, Chen R, Mukherjee N, Tong J, Kazmi S, Fang X, Witkowski MT, Aifantis I, et al. Bioengineered immunocompetent preclinical trial-on-chip tool enables screening of CAR T cell therapy for leukaemia. Nat Biomed Eng. 2025;9(12):2098–2114. 40595437 10.1038/s41551-025-01428-2 PMC12705464

[264] Murias-Closas A, Prats C, Calvo G, López-Codina D, Olesti E. Computational modelling of CAR T-cell therapy: From cellular kinetics to patient-level predictions. EBioMedicine. 2025;113: Article 105597. 40023046 10.1016/j.ebiom.2025.105597 PMC11914757

[265] Xiao Y, Li Y, Jing X, Weng L, Liu X, Liu Q, Chen K. Organoid models in oncology: Advancing precision cancer therapy and vaccine development. Cancer Biol Med. 2025;22(8):903–927. 40708272 10.20892/j.issn.2095-3941.2025.0127 PMC12418270

[266] Kang X, Cheemalamarri SK, Yin Q. Organoid: A promising solution to current challenges in cancer immunotherapy. npj Biomed Innov. 2025;2(1): Article 49. 10.1038/s44385-025-00051-9 PMC13055085 42032105

[267] Córdoba-Espejo L, Sánchez-Vega L, García-Ortiz A, Castellano E, Oliva R, Ortiz-Ruiz A, Gil-Alós D, Fernández A, Sanjurjo D, López-García S, et al. GMP-compliant manufacturing of allogeneic peripheral blood CAR-NK cells for the treatment of acute myeloid leukemia. Cytotherapy. 2025;28(2): Article 101968. 41386018 10.1016/j.jcyt.2025.07.009

[268] Balkhi S, Zuccolotto G, Di Spirito A, Rosato A, Mortara L. CAR-NK cell therapy: Promise and challenges in solid tumors. Front Immunol. 2025;16:1574742. 40260240 10.3389/fimmu.2025.1574742 PMC12009813

[269] Fang F, Xie S, Chen M, Li Y, Yue J, Ma J, Shu X, He Y, Xiao W, Tian Z. Advances in NK cell production. Cell Mol Immunol. 2022;19(4):460–481. 34983953 10.1038/s41423-021-00808-3 PMC8975878

[270] Saultz JN, Otegbeye F. Optimizing the cryopreservation and post-thaw recovery of natural killer cells is critical for the success of off-the-shelf platforms. Front Immunol. 2023;14:1304689. 38193082 10.3389/fimmu.2023.1304689 PMC10773738

[271] Yao X, Matosevic S. Cryopreservation of NK and T cells without DMSO for adoptive cell-based immunotherapy. BioDrugs. 2021;35(5):529–545. 34427899 10.1007/s40259-021-00494-7 PMC12376086

[272] Li W, Wang X, Zhang X, Aziz AUR, Wang D. CAR-NK cell therapy: A transformative approach to overcoming oncological challenges. Biomolecules. 2024;14(8): Article 1035. 39199421 10.3390/biom14081035 PMC11352442

[273] von Werz V, Szarzynski A, Zigon-Branc S, Spadiut O. Quality standards for NK cell immunotherapies. Front Bioeng Biotechnol. 2025;13:1716975. 41306902 10.3389/fbioe.2025.1716975 PMC12644036

[274] Wang B, Chen RQ, Li J, Roy K. Interfacing data science with cell therapy manufacturing: Where we are and where we need to be. Cytotherapy. 2024;26(9):967–979. 38842968 10.1016/j.jcyt.2024.03.011

[275] Kim J-H, Kawase E, Bharti K, Karnieli O, Arakawa Y, Stacey G. Perspectives on the cost of goods for hPSC banks for manufacture of cell therapies. npj Regener Med. 2022;7(1):54. 10.1038/s41536-022-00242-7 PMC9522845 36175440

[276] Thavorn K, Thompson ER, Kumar S, Heiskanen A, Agarwal A, Atkins H, Shorr R, Hawrysh T, Chan KK, Presseau J, et al. Economic evaluations of chimeric antigen receptor T-cell therapies for hematologic and solid malignancies: A systematic review. Value Health. 2024;27(8):1149–1173. 38641057 10.1016/j.jval.2024.04.004

[277] Adair JE, Anthony-Gonda K, Bayigga L, Orentas R, Mutuluuza CK, Mathews V, Dropulić B. Place-of-care manufacturing of gene therapies. Lancet Haematol. 2022;9(11):e807–e808. 36328038 10.1016/S2352-3026(22)00327-1

[278] Rouce RH, Grilley BJ. How to democratize cell and gene therapy: A global approach. Mol Ther. 2025;33(5):2082–2090. 40181548 10.1016/j.ymthe.2025.03.061 PMC12126836

